# Fusion with ARRDC1 or CD63: A Strategy to Enhance p53 Loading into Extracellular Vesicles for Tumor Suppression

**DOI:** 10.3390/biom14050591

**Published:** 2024-05-16

**Authors:** Min Liu, Yu Zhang, Jianfeng He, Wanxi Liu, Zhexuan Li, Yiti Zhang, Ao Gu, Mingri Zhao, Mujun Liu, Xionghao Liu

**Affiliations:** 1 Center for Medical Genetics & Hunan Key Laboratory of Medical Genetics, School of Life Sciences, Central South University, Changsha 410078, China; liumin@sklmg.edu.cn (M.L.); zhangyu@csu.edu.cn (Y.Z.); hejianfeng@sklmg.edu.cn (J.H.); liuwanxi@sklmg.edu.cn (W.L.); lizhexuan@xiangya.com.cn (Z.L.); zhangyiti@sklmg.edu.cn (Y.Z.); guao@sklmg.edu.cn (A.G.); zhaomingri@sklmg.edu.cn (M.Z.); 2 Department of Cell Biology, School of Life Sciences, Central South University, Changsha 410013, China; 3 Hunan Key Laboratory of Basic and Applied Hematology, Central South University, Changsha 410078, China; 4 Hunan Key Laboratory of Animal Models for Human Diseases, Central South University, Changsha 410078, China

**Keywords:** tumor, extracellular vesicles, p53, ARRDC1, CD63

## Abstract

**Simple Summary:**

This study investigates the potential of small extracellular vesicles (sEVs) as vehicles for delivering therapeutic agents, with a focus on the p53 tumor suppressor gene. Fusion with CD63 or ARRDC1 was found to facilitate the overexpression and proper localization of p53 and significantly increase the yield and loading efficiency of sEVs with p53 mRNA and proteins. Moreover, the functional assessment of these engineered sEVs on H1299 cells demonstrated an enhanced anti-tumor effect, particularly with ARP-sEVs, suggesting a superior inhibition of cell proliferation and promotion of apoptosis. These results are expected to be informative in the application of EV therapy.

**Abstract:**

Small extracellular vesicles (sEVs) have emerged as promising therapeutic agents and drug delivery vehicles. Targeted modification of sEVs and their contents using genetic modification strategies is one of the most popular methods. This study investigated the effects of p53 fusion with arrestin domain-containing protein 1 (ARRDC1) and CD63 on the generation of sEVs, p53 loading efficiency, and therapeutic efficacy. Overexpression of either ARRDC1–p53 (ARP) or CD63–p53 (CDP) significantly elevated p53 mRNA and protein levels. The incorporation of ARRDC1 and CD63 significantly enhanced HEK293T-sEV biogenesis, evidenced by significant increases in sEV-associated proteins TSG101 and LAMP1, resulting in a boost in sEV production. Importantly, fusion with ARRDC1 or CD63 substantially increased the efficiency of loading both p53 fusion proteins and its mRNA into sEVs. sEVs equipped with ARP or CDP significantly enhanced the enrichment of p53 fusion proteins and mRNA in p53-null H1299 cells, resulting in a marked increase in apoptosis and a reduction in cell proliferation, with ARP-sEVs demonstrating greater effectiveness than CDP-sEVs. These findings underscore the enhanced functionality of ARRDC1- and CD63-modified sEVs, emphasizing the potential of genetic modifications in sEV-based therapies for targeted cancer treatment.

## 1. Introduction

Extracellular vesicles (EVs) are naturally occurring membrane-bound vesicles re-leased into the extracellular environment for intercellular communication, encapsulating functional proteins and RNAs [1,2]. Small extracellular vesicles (sEVs) are characterized by a diameter of less than 200 nm with tetraspanins CD63, CD81, and CD9 present in their membrane [3]. The sEVs currently obtained from cell culture supernatants or liquid biopsy samples are inherently heterogeneous clusters of uncertain vesicles, including exosomes, arrestin domain-containing protein 1 (ARRDC1)-mediated microvesicles (ARMMs) and others [4,5]. Recent advancements have spotlighted sEVs as promising vehicles for delivering therapeutic agents, addressing a broad spectrum of diseases and showcasing their potential in clinical applications [6,7].

sEVs draw broad interest as nucleic acid or drug carriers owing to their excellent biocompatibility, low immunogenicity and because they are well tolerated [8,9]. EVs are membrane-permeable and can cross the blood–brain barrier (BBB) [10]. The presence of transmembrane and membrane-anchored proteins in sEVs prolongs blood circulation, facilitates tissue-directed delivery, and promotes cellular uptake of encapsulated contents [6,11]. Researchers investigated various methods, such as mechanical extrusion, hypoxia induction, and 3D culture, to upscale sEV production and enhance drug loading capacity [12,13]. For genetic modification strategies, numerous studies have fused target genes directly to membrane proteins of sEVs to encapsulate target molecules into sEVs and increase the production of sEVs. Reported proteins used for these studies include exosome-associated proteins such as LAMP2, LMP1, CD63, CD9 and the ARMM-associated protein ARRDC1 [14,15,16,17]. Based on their study settings, researchers often choose one of these proteins for subsequent studies, and no comparative studies on the expression and function of target genes after fusing different membrane proteins has been made available so far.

EVs are an advanced and novel category of therapeutic agents that may serve as substitutes for cell therapies [18]. A statistic in 2020 revealed that 157 exosome-related clinical trials had been registered with clinicaltrials.gov [19]. As drug carriers, EVs deliver encapsulated cargo to both adjacent and distant cells, regulating gene expression and modifying the phenotypes of recipient cells [14,20]. However, EV-based drug delivery currently faces challenges, including limited drug loading efficiency and insufficient clinical-grade production [21,22]. Enhancing exosome production without compromising loading efficiency is achievable through genetic manipulation of the critical genes involved in exosome biosynthesis and recycling, such as LMP1, LMP2, CD63, and CD9 [23,24,25]. Additionally, ARRDC1 has been shown to increase the yield of sEVs and assist in the packaging and delivery of mRNA and protein into ARMMs to target cells [17]. Despite identifying various supportive molecules, the lack of a systematic comparison of their capacities to improve yield, loading efficiency, and therapeutic efficacy has left researchers uncertain about their application.

Mutations in the TP53 gene are often accompanied by tumorigenesis. Loss-of-function p53 tumor suppressor mutations can result in inefficient DNA base excision repair, loss of cell cycle arrest, loss of apoptosis, and loss of senescent growth arrest, thereby supporting cancer proliferation [26]. Delivering functional p53 represents a viable therapeutic strategy for many cancers with compromised p53 function. In this study, we explored how CD63 and ARRDC1, as components of fusion proteins, enhance exosome production and selectively incorporate co-expressed p53 into small extracellular vesicles (sEVs) and then assessed these sEVs’ apoptotic and proliferation-inhibiting effects on p53-null H1299 cells. The present research will pave the way for the strategic application of genetic modifications in sEV-based therapies.

## 2. Materials and Methods 

### 2.1. Plasmid Construction

This study used minipHrneo as an expression vector containing a cytomegalovirus promoter-driven expression cassette [27]. The p53 expression construct was generated by integrating a full-length DNA fragment of wild-type TP53 between the PacI and MfeI sites of the minipHrneo vector. For the construction of the ARRDC1–p53 (ARP) fusion construct, the entire sequences of ARRDC1 and wild-type TP53 DNA were amplified by PCR and then incorporated into the minipHrneo vector using the ClonExpress^®^ MultiS One Step Cloning Kit (Vazyme, Nanjing, China). The CD63–p53 (CDP) expression construct was obtained in similar fashion. Primer sequences utilized are listed in Supplementary Table S1. PCR and DNA sequencing were used to verify all constructs (Supplementary Figure S1). Restriction endonucleases were procured from New England Biolabs (Beverly, MA, USA), while PCR reaction kits and ligation kits were sourced from Vazyme and Takara Bio (Shiga, Japan), respectively.

### 2.2. Cell Culture and Transfection

The human embryonic kidney 293T(HEK293T) cell line was purchased from the ATCC and maintained in Dulbecco’s modified Eagle’s medium (Gibco, New York, NY, USA) supplemented with 10% fetal bovine serum (FBS) (Gibco, Carlsbad, CA, USA). The H1299 cell line was gifted to us from the Institute for Advanced Study of Central South University and was cultured in RPMI-1640 medium (Gibco, New York) supplemented with 10% FBS. Both cell lines were incubated at 37 °C with 5% CO_2_ and regularly checked for mycoplasma contamination. HEK293T and H1299 cells were transfected using Lipofectamine 2000 reagent (Invitrogen, Carlsbad, CA, USA) and OPTI-MEM (Gibco, New York) according to the manufacturer’s protocol. After 6 h, the transfection mixtures were replaced with fresh medium. Cells or supernatants were collected 48 h post-transfection for further analysis.

### 2.3. sEV Purification

Following transfection of various constructs into HEK293T or H1299 cells, cells were washed thrice with DPBS after 6 h, and the media was replaced with fresh DMEM or RPMI-1640 supplemented with 10% EXO-depleted FBS (VivaCell, Shanghai, China). Following 48 h post-transfection, the cell culture supernatant was collected and centrifuged for three rounds in sequence (300× *g* for 10 min, 3000× *g* for 30 min and 10,000× *g* for 30 min). The supernatant was then filtered through a 0.22 μm pore filter (EMD Millipore, Billerica, MA, USA) to remove larger particles. Subsequently, the supernatants were transferred to an ultrafiltration tube with a 100 kDa centrifuge concentrator (EMD Millipore) and concentrated at 3000× *g* for 30 min at 4 °C. Finally, sEVs were then isolated by enrichment with ExoQuick-TC™ (System Biosciences, Inc., Palo Alto, CA, USA). The supernatant was mixed with equal volumes of the ExoQuick-TC™ solution and refrigerated at 4 °C overnight for at least 12 h. Following centrifugation at 1500× *g* for 30 min at 4 °C, the supernatant was discarded, and the pellet containing sEVs was resuspended with DPBS and stored at −80 °C.

### 2.4. Biophysical Characterization and Imaging

Zetaview equipment PMX 110 (Particle Metrix, Meerbusch, Germany) was used to track the concentration and diameter of sEVs. Before nanoparticle tracking analysis (NTA), we diluted the samples with DPBS to a proper concentration and loaded 100 µL of the samples into the EV analysis chamber. For each set of measurements, three 30-s videos were recorded. Data obtained from Zetaview 8.04.02 SP2 were processed using Microsoft Excel 2019 (Microsoft Corp., Seattle, WA, USA). Cell counts were conducted for each plate to normalize the concentration of sEVs.

In preparation for imaging via negative staining transmission electron microscopy (TEM), sEVs were suspended in 20 μL of DPBS. Droplets of this suspension were deposited onto carbon-coated 200 mesh copper grids and allowed to adhere for over 1 min. Subsequently, the grids were treated with a 2% uranyl acetate solution for 10 min to facilitate negative staining, with excess fluid absorbed using filter paper. Micrographs were then obtained using a Tecnai biological transmission electron microscope (model: Tecnai G2 Spirit, Thermo Fisher Scientific, Waltham, MA, USA) at 80 KV.

### 2.5. Immunoblot Analysis and Antibodies

Cell or EV proteins were extracted using RIPA Lysis Buffer (Beyotime, Shanghai, China) and a Protease Inhibitor Cocktail (Sigma-Aldrich, St. Louis, MO, USA). Protein concentrations were quantified using a Pierce™ BCA Protein Assay Kit (Thermo Fisher Scientific). 

For the detection of p53, TSG101, and LAMP1, 6 µg of cell lysate or 30 µg of sEV lysate samples were loaded onto a 10% SDS-PAGE (Yeasen, Shanghai, China) and transferred to polyvinylidene difluoride (PVDF) membrane (Millipore, Bedford, MA, USA). After blocking with 5% skim milk in 1 × TBST (TBS, 0.1%Tween 20) buffer for 1 h, membranes were incubated with the primary antibodies: mouse p53 (1:500, SC-126; Santa Cruz, CA, USA), rabbit tumor susceptibility gene 101 (TSG101) (1:1000, R25999; zenbio, Chengdu, China), rabbit lysosome-associated membrane glycoprotein 1 (LAMP1) (1:1000, 9091S; Cell signaling, Trask Lane, Danvers, MA, USA), mouse GAPDH (1:1000, SC-32233; Santa Cruz), and mouse β-actin (1:10,000, A1978; Sigma-Aldrich), respectively, at 4 °C overnight. The next day, membranes were washed three times with 1 × TBST and incubated with the horseradish peroxidase-conjugated anti-mouse secondary antibodies (1:10 000; Jackson ImmunoResearch, West Grove, PA, USA) and anti-rabbit (1:10,000; Jackson ImmunoResearch) for 1.5 h at room temperature, correspondingly. Protein signals were detected using an enhanced chemiluminescence kit (Super-Signal™ West Femto Maximum Sensitivity Substrate, Thermo Fisher Scientific) and imaged with a Bio-Rad imaging system (Bio-Rad, Hercules, CA, USA). Pre-stained molecular weight standards (Thermo Fisher Scientific) were used for molecular weight estimation.

To detect EV markers, 30 µg of protein lysate was loaded onto 10% SDS-PAGE gels. After blocking, PVDF membranes were incubated with the primary antibodies against CD63 (1:500, SC-5275; Santa Cruz), CD9 (1:500, SC-13118; Santa Cruz), TSG101 (1:500, SC-7964; Santa Cruz), and Calnexin (1:500; SC-13118, Santa Cruz) overnight at 4 °C. Following washes, membranes were incubated with anti-mouse secondary antibodies (1:10,000; Jackson ImmunoResearch), and the blots were visualized using a gel imaging system.

### 2.6. Immunofluorescence Labeling and Confocal Imaging

Cells grown on glass coverslips underwent triple washes with DPBS prior to fixation with 4% paraformaldehyde for 15 min. After fixation, cells were permeabilized with 0.1% Triton X-100 in PBS for 5 min and washed with DPBS. Blocking was performed with 5% bovine serum albumin (BSA) for 1 h. Primary antibodies against mouse p53 (1:200, SC-126; Santa Cruz), mouse CD63 (1:200, SC-5275; Santa Cruz), rabbit ARRDC1 (1:200, ab181758; Abcam, Cambridge, MA, USA), and rabbit LAMP1 (1:200, 9091S; Cell signaling) were diluted in 5% BSA in PBS and incubated with the cells overnight at 4 °C. Following primary antibody incubation, cells were washed three times with DPBS and incubated with Alexa Fluor 488- or 555-conjugated secondary antibodies (1:250, Invitrogen), appropriate for the primary antibody host species. After staining with DAPI (Sigma-Aldrich) for 5–8 min and washing with water, the glass coverslips were affixed on glass slides. Images were captured using a LAS X SP-5 confocal microscope (Leica, Wetzlar, Germany).

### 2.7. Labeling sEVs and Tracking Cellular Uptake

sEVs were labeled with PKH67 fluorescent dye (Sigma-Aldrich) by incubation at a concentration of 5 μM at 37 °C for 10 min. The staining was terminated by mixing with 1 mL of 0.5% BSA, followed by transfer of the mixture to a 100 kDa centrifugal concentrator (EMD Millipore) and centrifugation at 3000× *g* for 30 min at 4 °C to remove unbound dye. After rinsing twice with 2–3 mL of DPBS to ensure complete removal of free dye, ExoQuick-TC™ (System Biosciences, Inc.) was added to precipitate the sEVs. Following refrigeration at 4 °C overnight for at least 12 h and centrifugation at 1500× *g* for 30 min at 4 °C, the sEVs were resuspended in 100 μL of basal medium and stored at −80 °C, protected from light.

For the in vitro uptake experiment, PKH67-labeled sEVs were incubated with H1299 cells (~30% confluence) at 37 °C for 6 h (final concentration of ~6 × 10^8^ Particles/mL). Then, cells were washed three times with cold DPBS and fixed in 4% paraformaldehyde solution for 10 min at room temperature. The nuclei were stained with DAPI for 8 min at room temperature, followed by two washes with DPBS. Image acquisition was performed using confocal fluorescence microscopy (Leica). 

### 2.8. Real-Time Quantitative Polymerase Chain Reaction

Total RNA was extracted from cells and sEVs using TRIzol reagent (Invitrogen). Complementary DNA (cDNA) was synthesized using the HiScript III First Strand cDNA Synthesis Kit (Vazyme). Expression levels of target genes, both in cells and sEVs, were quantified and normalized against GAPDH as an internal control. Data analysis was conducted using Bio-Rad CFX Manager software 3.1. Each PCR reaction was performed in triplicate. Primer sequences are provided in Supplementary Table S1.

### 2.9. Cell Viability Assay

H1299 cells were seeded at 6000 cells per well in a 96-well plate and adhered overnight. After washing twice with DPBS, HEK293T-sEVs input was added in fresh EXO-depleted medium for a final concentration of 1.2 to 1.5 × 10^10^ Particles/mL. After incubating for approximately 3 days, cell viability was assessed using a 10% CCK-8 solution (Vazyme). Absorbance was measured at 450 nm using an Agilent BioTek ELx800 microplate reader (BioTek, Winooski, VT, USA). Each assay was performed at least in triplicate.

### 2.10. Apoptosis Detection

To assess the pro-apoptotic ability of the constructs, H1299 cells were collected 48 h after transfection. Additionally, to evaluate the apoptotic induction capability of different sEVs, H1299 cells were harvested after 72 h of incubation with various sEVs at 37 °C and 5% CO_2_. Cells from each group were stained with Annexin V-FITC/PI Apoptosis Detection Kit (Vazyme). After washing twice with DPBS and resuspending in 1× Binding Buffer, cells were stained with 5 μL of Annexin V-fluorescein isothiocyanate (Annexin V-FITC) and 5 μL of propidium iodide (PI) and incubated in the dark for 10 min. Flow cytometry analysis was conducted within 1 h to estimate the percentage of apoptotic cancer cells. The data were analyzed using FlowJo software 10.0.7 (FlowJo, LLC, Ashland, OR, USA).

### 2.11. Statistical Analysis

Data processing and statistical analysis were performed using GraphPad Prism 8.3. Results are presented as the mean ± standard error of three samples unless otherwise stated. Statistical analysis was performed using Student’s *t*-test, with *p* values < 0.05 considered statistically significant.

## 3. Results

### 3.1. Expression, Function, and Localization of p53 Fusion Proteins

Constructs for p53, ARRDC1–p53 (ARP), and CD63–p53 (CDP), were developed with ARP and CDP being fusion constructs where ARRDC1 and CD63 are fused to the N-terminal end of wild-type TP53, respectively (Supplementary Figure S1). To assess whether fusing ARRDC1 or CD63 affects p53’s function, we transfected p53-null H1299 cells with vectors carrying the ARP or CDP expression cassettes. Cells transfected with a vector carrying p53 served as a positive control, while those with an empty vector were the negative control. Western blot analysis confirmed the successful expression of ARP or CDP fusion proteins in H1299 cells (Supplementary Figure S2A,B). Flow cytometry, conducted 48 h post-transfection, showed that both ARP and CDP fusion proteins promoted apoptosis in H1299 cells (Supplementary Figure S2C). 

**Figure 1:**
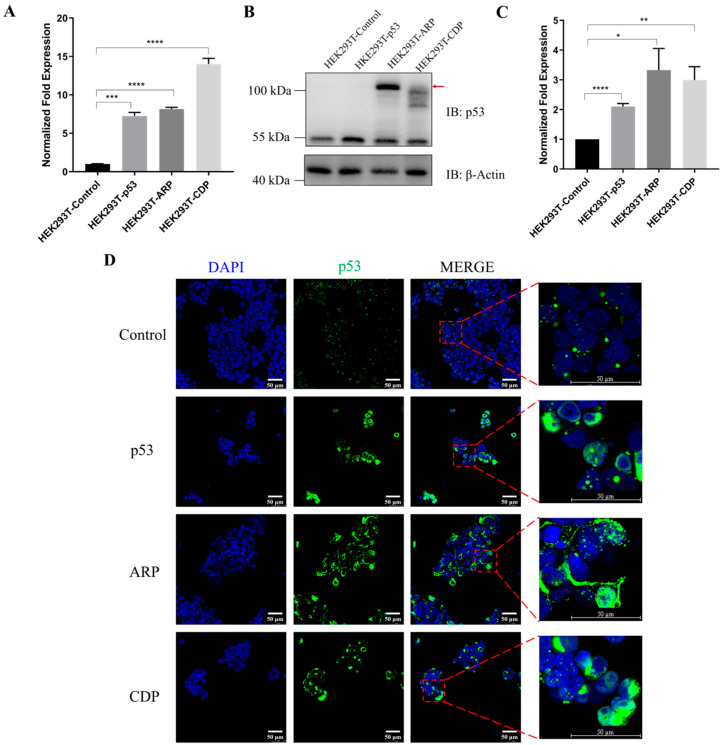
The expression and localization of p53 and its fusions ARP and CDP in HEK293T cells. (**A**) RT-qPCR analysis of p53 mRNA levels in HEK293T cells transfected with the control vector (minipHrneo), p53, ARP (ARRDC1–p53), and CDP (CD63–p53) (*n* = 3 per group; *** *p* < 0.001, **** *p* < 0.0001). (**B**,**C**) The expression levels of p53 protein in HEK293T cells were detected by Western blot analysis. The red arrow indicates the bands corresponding to fusion proteins. The expression levels of p53 and its fusion proteins were quantified by summing the intensities of all observed bands detected, normalized to β-actin. Note: Specific bands at ~55 kDa represent p53 protein; a band at ~110 kDa and additional lower smearing bands in the ARP group indicate ARRDC1–p53 fusion protein; a diffuse band at ~100 kDa in the CDP group represents the CD63–p53 fusion protein (*n* = 3 per group; * *p* < 0.05, ** *p* < 0.01, **** *p* < 0.0001). Original images can be found in Supplementary Materials. (**D**) Immunofluorescence microscopy showing the expression and subcellular localization of p53 and its fusions, ARP and CDP, in HEK293T cells. Nuclei are stained with DAPI (Blue). Scale bar = 50 µm.

Subsequently, HEK293T cells were transfected with the same set of vectors, generating HEK293T-derived sEVs for use as molecular transport carriers. Expression analysis revealed that p53 mRNA levels in the p53, ARP and CDP groups increased by approximately 7.25-fold, 8.16-fold, and 14.02-fold, respectively, compared to the control group (Figure 1A). Further, the expression of p53 protein was confirmed, as depicted in Figure 1B, with specific and smeared bands being observed for ARP or CDP fusion proteins. The smeared bands in both the ARP and CDP groups are presumably the results of the ubiquitination of ARRDC1 and CD63 [5,28]. Western blot and immunofluorescence analysis revealed a marked increase in the p53 protein level in cells transfected with p53, ARP, or CDP compared to those transfected with the empty vector (Figure 1C,D). Immunofluorescence observations for either the ARP or CDP group revealed an enhanced nuclear localization signal, suggesting that p53, when fused with either ARRDC1 or CD63, still retains its capability for nuclear entry (Figure 1D and Supplementary Figure S3). 

### 3.2. ARRDC1 and CD63 Enhance ARMMs and Exosome Gene Expression

**Figure 2:**
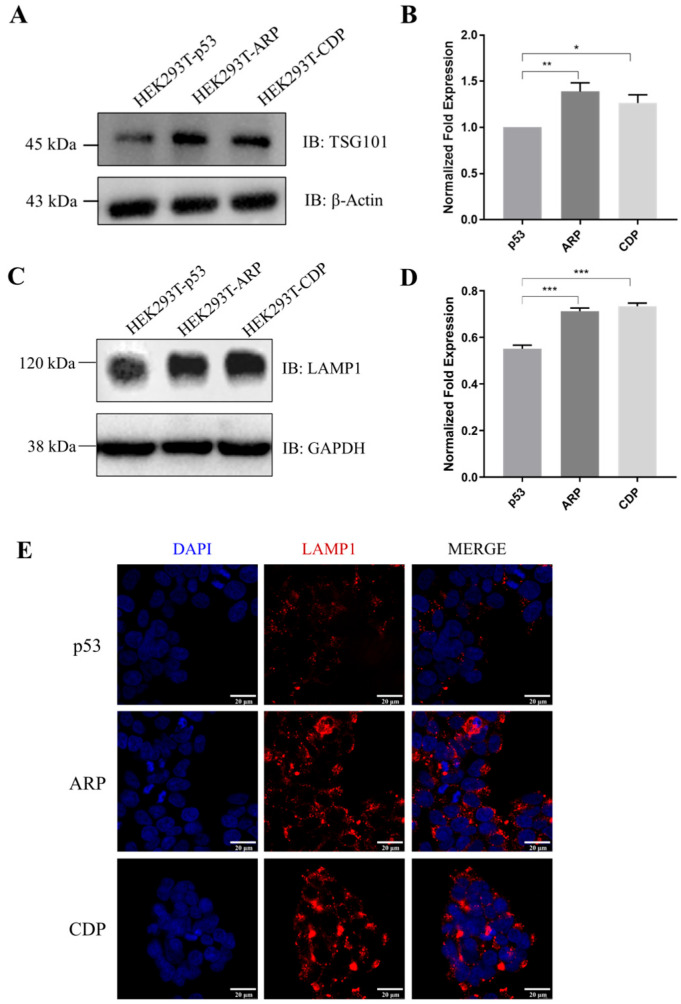
Analysis of TSG101 and LAMP1 protein expression in HEK293T cells post-transfection. (**A**,**B**) Western blot analysis showing the expression levels of TSG101 protein in HEK293T cells transfected with expression constructs for p53, ARP, or CDP, normalized to β-actin (*n* = 3 per group; * *p* < 0.05, ** *p* < 0.01). (**C**,**D**) Western blot analysis showing the expression levels of TSG101 protein in HEK293T cells transfected with expression constructs for p53, ARP, or CDP, normalized to GAPDH (*n* = 3 per group; *** *p* < 0.001). Original images can be found in Supplementary Materials. (**E**) Immunofluorescence staining illustrating the expression and localization level of the late endosomal marker LAMP1 (Red), with cell nuclei stained using DAPI (Blue). Compared to the p53 group, cells transfected with the expression constructs for ARP or CDP showed enhanced LAMP1 fluorescence signals. Scale bar = 20 µm.

To examine whether ARP and CDP could affect the formation of sEVs, such as ARMMs or exosomes, we analyzed the expression of related proteins in HEK293T cells transfected with p53, ARRDC1–p53, or CD63–p53 constructs. The specific interaction between TSG101 and ARRDC1 drives the budding and releasing of ARMMs [5]. Additionally, as an essential component of ESCRT-I, TSG101 plays a significant role in the incorporation and sorting of cargoes into MVBs, influencing the formation of exosomes [29,30]. Lysosome-associated membrane protein 1 (LAMP1) is a specific surface protein for the late endosomal stage of exosome formation within the cell [31]. Our results show an increased abundance of TSG101 and LAMP1 in cells transfected with CD63–p53, compared to those transfected with the p53 construct (Figure 2A–D). Notably, in addition to confirming that ARRDC1 promoted the expression of TSG101, we also demonstrated that the overexpression of ARRDC1 led to an increased expression of LAMP1 by comparing the ARP and p53 groups (Figure 2A–E).

### 3.3. Improved sEV Production and p53 Loading Efficiency by ARP or CDP Transfection

**Figure 3:**
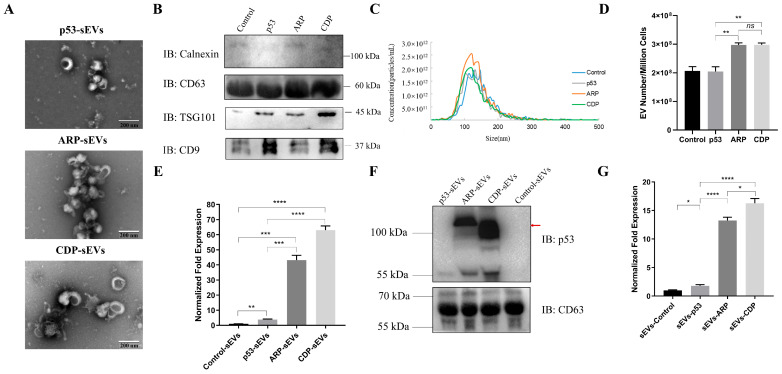
Characterization of HEK293T-derived sEVs and enrichment analysis of p53 and its fusions in sEVs. (**A**) Electron micrographs display the characteristic cup-shaped and bilayered membrane structure of sEVs derived from HEK293T cells transfected with p53, ARP, or CDP expression constructs. Scale bar = 200 nm. (**B**) Western blot confirming the presence of EV marker proteins (CD63, TSG101, CD9) and the absence of cellular protein calnexin in sEVs from the different groups. sEVs from HEK293T cells transfected with the control vector (minipHrneo) served as controls. (**C**) The representative size distribution of HEK293T-sEVs from the different groups. (**D**) Quantitative analysis of HEK293T-derived sEVs produced per million cells across different groups (*n* = 3 per group, ** *p* < 0.01, *ns*, not significant). (**E**) RT-qPCR analysis of mRNA levels for p53 and its fusions in sEVs, indicating significantly higher levels in sEVs from the ARP and CDP groups compared to the p53 groups (*n* = 3 per group; ** *p* < 0.01, *** *p* < 0.001, **** *p* < 0.0001). (**F**,**G**) Western blot analysis revealed markedly elevated fusion protein loading levels in the ARP and CDP groups compared to the p53 group. The red arrow highlights bands corresponding to fusion proteins. Quantification was performed by summing the intensities of all observed bands detected for p53, normalized against CD63 (*n* = 3 per group; * *p* < 0.05, **** *p* < 0.0001). Original images can be found in Supplementary Materials.

Following transfection, sEVs were isolated from the HEK293T cells employing an optimized isolation method. TEM revealed that these samples primarily exhibited a near-spherical shape with a bilayered membrane structure. Their diameter was within the anticipated size range for 50–200 nm (Figure 3A). The identity of these sEVs was further confirmed by the presence of TSG101, CD9, and CD63, key sEV markers, and the absence of the cellular protein marker calnexin (Figure 3B). Additionally, NTA revealed that the size distributions of all groups of samples were consistent with the characteristics of sEVs (Figure 3C). No difference in the sizes of the sEVs was found, with mean sizes of 129.4 nm, 129.5 nm, 127.4 nm, and 127.8 nm in the control, p53, ARP, and CDP groups, respectively (Supplementary Figure S4). Notably, the quantity of sEVs significantly increased in cells transfected with ARP and CDP, showing a 1.47-fold and a 1.44-fold enhancement, respectively, compared to those transfected with p53 (Figure 3D).

To determine whether ARP or CDP specifically enhance the incorporation of cargo into sEVs, we measured p53 mRNA and protein levels within sEVs. Results presented in Figure 3E indicate that sEVs from cells transfected with ARP or CDP showed increased p53 mRNA levels by 11.2-fold and 16.3-fold, respectively, compared to those from the p53 group. Similarly, a significant enrichment of p53 fusion proteins in sEVs from ARP and CDP groups was observed (Figure 3F). Furthermore, we assessed the loading efficiency of p53 and its fusion proteins into the sEVs across four groups, normalized against CD63 [32]. The comparison revealed that the loading efficiency in the ARP and CDP groups was 6.34 and 8.03 times higher, respectively, than in the p53 group (Figure 3G). Collectively, these results imply that ARRDC1 and CD63 facilitate the selective enrichment of p53 fusion proteins and their mRNA into sEVs, leading to increased loading efficiency.

### 3.4. ARP-sEVs Outperform CDP-sEVs in Anti-Tumor Effects on H1299 Cells

**Figure 4:**
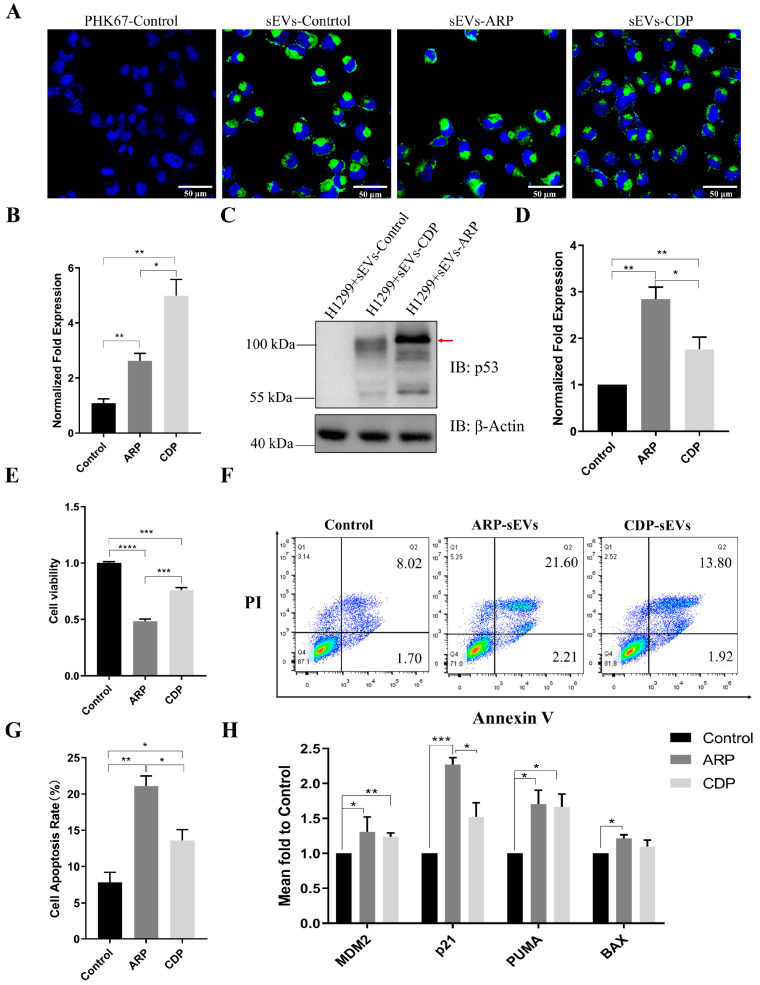
ARP-sEVs and CDP-sEVs inhibit growth and promote apoptosis of p53-null H1299 cells in vitro. (**A**) Microscopic analysis showing the uptake of sEVs by H1299 cells, with nuclei stained by DAPI (Blue) and sEVs marked by PKH67 (Green). PKH67-control were prepared using DPBS mixed with dye, following the same steps of the other groups. Scale bar = 50 µm. (**B**) RT-qPCR analysis of mRNA levels for p53 and its fusion products in H1299 cells co-cultured with different sEVs (*n* = 3 per group; * *p* < 0.05, ** p < 0.01). (**C**,**D**) Western blot analysis of p53 and its fusion proteins in H1299 cells treated with different sEVs. The red arrow highlights the bands corresponding to fusion proteins. Quantification was performed by summing the intensities of all observed bands detected in IB for p53, normalized against β-actin (*n* = 3 per group; * p < 0.05, ** p < 0.01). Original images can be found in Supplementary Materials. (**E**) Cell viability assays (CCK8) showed a decreased viability in the H1299 cells treated with ARP-sEVs and CDP-sEVs compared to the controls (*n* = 3 per group; *** p < 0.001, **** p < 0.0001). (**F**,**G**) Flow cytometric analysis and statistical data showing increased apoptosis in H1299 cells co-cultured with ARP-sEVs and CDP-sEVs (*n* = 3 per group; * p < 0.05, ** p < 0.01). (**H**) RT-qPCR analysis of p53 target genes (MDM2, p21, PUMA, BAX) in H1299 recipient cells co-cultured with different sEVs (*n* = 3 per group; * p < 0.05, ** *p* < 0.01, *** p < 0.001).

sEVs derived from HEK293T cells, labeled with PKH67 dye (green), were confirmed to be taken up by H1299 cells using confocal microscopy after incubation (Figure 4A). To assess the anti-proliferative and pro-apoptotic effects of ARP and CDP loaded into sEVs on H1299 cells, sEVs loaded with ARP and sEVs loaded with a corresponding amount of CDP, normalized against p53 protein levels, were incubated with H1299 cells for 48 h. Expression analysis saw a significant increase of p53 mRNA and protein levels in H1299 cells following ARP-sEVs or CDP-sEVs treatment (Figure 4B–D). Moreover, ARP-sEVs induced a greater increase in p53 and its fusion protein levels than CDP-sEVs, as depicted in Figure 4C. Cell viability assays revealed that both ARP-sEVs and CDP-sEVs significantly inhibited H1299 cell proliferation, with ARP-sEVs being more effective (Figure 4E). Apoptosis assays further showed that ARP-sEVs and CDP-sEVs markedly increased the apoptotic rates in H1299 cells beyond those observed with sEVs from HEK293T cells transfected with a control empty vector, especially with ARP-sEVs showing greater efficacy (Figure 4F,G). Additionally, the uptake of ARP-sEVs or CDP-sEVs led to an enhanced expression of MDM2, p21, PUMA, and BAX in H1299 cells, with a marked increase in p21 expression observed in the ARP-sEVs-treated group compared to the CDP-sEVs group (Figure 4H). These results indicated the superior efficacy of ARP-sEVs over CDP-sEVs in inhibiting proliferation and enhancing apoptosis in H1299 cells.

## 4. Discussion

EVs have emerged as an innovative drug delivery system, efficiently transporting encapsulated substances to adjacent and distant cells to modulate gene expression and alter phenotypes of recipient cells [19,20,21]. ARRDC1 enhances ARMM production and their content delivery, including mRNA and proteins, to target cells [17]. Moreover, genetic alterations in essential components of the exosome biosynthesis pathways, such as LMP1, LMP2, CD63, and CD9, have shown potential in increasing exosome production without sacrificing loading efficiency [23,24,25]. In this study, we leveraged the anti-tumor properties of the p53 protein to investigate and compare the effectiveness of ARRDC1–p53 (ARP)-sEVs and CD63–p53 (CDP)-sEVs. By constructing p53, ARP, and CDP vectors, we explored the expression, function, and localization of p53 proteins and their effects on the formation of sEVs and the modulation of H1299 cell activities. Our findings reveal a 50% increase in sEV production from cells overexpressing ARRDC1–p53 and CD63–p53, alongside an improved efficiency in the loading of p53 molecules into ARP-sEVs and CDP-sEVs. Furthermore, we have demonstrated that ARP-sEVs and CDP-sEVs not only effectively deliver p53 but also significantly inhibit proliferation and induce apoptosis in H1299 lung cancer cells in vitro, with ARP-sEVs exhibiting superior anti-tumor efficacy compared to CDP-sEVs. These results underscore the potential of ARRDC1–p53 overexpressing sEVs as a viable p53 delivery platform for anti-cancer research.

Vesicle biogenesis and secretion is a complex process carefully regulated and controlled by several genes including SNARE, TSG101, VPS4, and RAB7 [5,33,34]. Exosomes and ARMMs, as distinct sEV subtypes, occupy the 50–200 nm size range but are differentiated by their specific biogenetic pathways [35]. It was well documented that ARRDC1 facilitates the budding of ARMMs by engaging TSG101 [5]. Our study reveals that cells overexpressing ARRDC1–p53 significantly increase sEV production, accompanied by elevated TSG101 levels in the donor cells (Figure 2A,B and Figure 3D). Intriguingly, a novel observation from our research is that ARRDC1–p53 overexpression also leads to the formation of late endosomes marked by LAMP1 (Figure 2C–E), hinting at ARRDC1’s broader involvement in exosome generation. This underscores the importance of further research into ARRDC1’s roles in vesicle dynamics, providing fresh insights into the molecular regulatory functions of ARRDC1 and highlighting its potential in sEV-based applications.

Several studies have shown that cell-derived sEVs designed with overexpression vectors are significantly enriched for specific mRNAs and proteins. For instance, J.L. Yang et al. identified a significant increase in NGF mRNA within exosomes from NGF-overexpressing HEK293 cells [36]. Similarly, F. Li et al. observed a 3–5-fold rise in BMP2 mRNA levels in MSC-BMP2-Exo post-BMP2 transfection [37]. Moreover, Villamizar O. et al. detected a notable accumulation of CFZF-VPR protein in sEVs of MSCs and HEK293 cells transfected with CFZF-VPR, which activated CFTR transcription in recipient cells after delivery by sEVs [38]. Mirroring these discoveries, our study demonstrated that p53 overexpression substantially enhanced its encapsulation rate within sEVs. Remarkably, the co-overexpression of ARRDC1–p53 and CD63–p53 led to a more efficient enrichment of p53 molecules in sEVs. In our work, we found an enrichment of p53 mRNA in sEVs derived from cells overexpressing p53 up to 3.86 times that of the control group, while in ARP-sEVs and CDP-sEVs, the enrichment levels of p53 mRNA were 11.20 and 16.34 times higher than those in p53-sEVs, respectively (Figure 3E). The enrichment of p53 protein in p53-sEVs, ARP-sEVs, and CDP-sEVs was increased to 1.80, 13.24, and 16.28 times that of the control group, respectively (Figure 3G). These findings affirm the sEVs’ exceptional capacity as an efficient vehicle for delivering therapeutic molecules, highlighting their potential in advancing gene therapy and precision medicine.

In this study, three members were utilized to construct fusion proteins, each localized distinctly. p53 possesses nuclear localization and export signal sequences, enabling its continuous transport between the nucleus and the cytoplasm [39]. ARRDC1 is anchored to the cytosolic side of the plasma membrane [5], whereas CD63 predominantly resides in the cytoplasm with a partial presence on the cell membrane [40]. Our experiments revealed enhanced nuclear localization signals when p53, ARP, or CDP were overexpressed in HEK293T cells, highlighting the functional activity of p53 within the nucleus. Significant differences in localization patterns among ARP and CDP were noted, with ARP and CDP showing pronounced accumulation in the cell membrane and cytoplasm, respectively (Figure 1D). Concurrently, similar localization patterns were confirmed by additional experiments depicted in Figure S3. These findings suggest that the distinct aggregation patterns of the p53 fusion proteins are influenced by the native localizations of ARRDC1 and CD63. This effect likely impacts the loading efficiency of target protein into ARP-sEVs and CDP-sEVs, as evidenced by the increased accumulation of ARP and CDP in these vesicles (Figure 3E–G). Although a significant enrichment of p53 was observed in both ARP-sEVs and CDP-sEVs, their anti-tumor activities varied (Figure 4E–G); the variation in their anti-tumor effects likely stems from differences in the recipient cells’ p53 uptake and processing post-sEV delivery. Investigating the behavior of ARRDC1–p53 and CD63–p53 in sEVs is thus critical. Developing components that can be selectively disengaged from the ARRDC1–p53 and CD63–p53 constructs could markedly optimize sEV functionality.

## 5. Conclusions

In conclusion, our study highlights the application of ARRDC1–p53 (ARP) and CD63–p53 (CDP) fusion constructs in augmenting the functionality of cell-derived sEVs, showcasing enhanced p53 mRNA and protein enrichment in sEVs, alongside superior anti-tumor efficacy. These constructs boosted sEV generation and markedly enhanced p53 mRNA and protein loading efficiency in sEVs. Both ARP-sEVs and CDP-sEVs could effectively deliver p53 and significantly inhibit the proliferation and induce apoptosis of H1299 lung cancer cells, with ARP-sEVs demonstrating notably superior anti-tumor properties than CDP-sEVs. These results suggest that both ARRDC1 and CD63 could serve as promising auxiliary genes for the sEV-mediated delivery of therapeutic targets in the future, with ARRDC1 showing even greater potential for application. This study enriches our understanding of sEVs in molecular transport and sets the foundation for sophisticated targeted delivery systems.

## Data Availability

The data used in this study are available from the corresponding author upon reasonable request.

## Supplementary Materials

The following supporting information can be downloaded at: https://www.mdpi.com/article/10.3390/biom14050591/s1, Table S1: Primers in the present study involved; Figure S1: Validate constructions by PCR and Sanger sequences; Figure S2: Analysis of p53 protein expression and apoptotic effects in H1299 cells; Figure S3: The location of ARRDC1 and CD63 in different groups of HEK293T cells; Figure S4: Diameter (median) of sEVs in nm measured by NTA.
