# Supplementary material for: Fusion with ARRDC1 or CD63: A Strategy to Enhance p53 Loading into Extracellular Vesicles for Tumor Suppression
Source: Biomolecules. 2024 May 16;14(5):591. doi: 10.3390/biom14050591 (PMC11118238; doi:10.3390/biom14050591)
Supplement: Supplementary file 1 [file biomolecules-14-00591-s001.zip › Sup_ARP/~WRL1483.tmp]

**SUPPLEMENTAL MATERIAL**

**SUPPLEMENTAL TABLE 1.** Primers in the present study involved.

|  | **Primer** | **Sequence (5’-3’)** |
| --- | --- | --- |
| Primers for P53 construction | P53-F | ttaattaaATGGAGGAGCCGCAGTCAGAT |
|  | P53-R | caattgTCAGTCTGAGTCAGGCCCTTCT |
| Primers for ARP construction | 1-phrn-AR-F1 | ccgggatccccgcggttaattaaATGGGGCGAGTGCAGCTC |
|  | 2-ARP-R1 | ggctcctccatTAAGACGGTGGGGCCTCTG |
|  | 3-ARP-F2 | accgtcttaATGGAGGAGCCGCAGTCAG |
|  | 4-P-phrn-R2 | tgcaataaacaagttcaattgTCAGTCTGAGTCAGGCCCTTCT |
| Primers for CDP construction | 1-phrn-CD-F1 | ccgggatccccgcggttaattaaATGGCGGTGGAAGGAGGA |
|  | 2- CDP-R1 | atCACCTCGTAGCCACTTCTGATACTC |
|  | 3- CDP-F2 | agaagtggctacgaggtgATGGAGGAGCCGCAGTCAG |
|  | 4-P-phrn-R2 | tgcaataaacaagttcaattgTCAGTCTGAGTCAGGCCCTTCT |
| Primers for validating constructs | pretarget-F | GCAGAGCTGGTTTAGTGAA |
|  | LHA-R | GTTCACCTACGGAAACCTT |
| Primers for P53 qPCR | qP53-F | CCTGAGGTTGGCTCTGACTG |
|  | qP53-R | CACGCACCTCAAAGCTGTTC |
| Primers for apoptosis related molecules | qp21-F | TGTCCGTCAGAACCCATGC |
|  | qp21-R | AAAGTCGAAGTTCCATCGCTC |
|  | qMDM2-F | GAATCATCGGACTCAGGTACATC |
|  | qMDM2-R | TCTGTCTCACTAATTGCTCTCCT |
|  | qPUMA-F | GACCTCAACGCACAGTACGAG |
|  | qPUMA-R | AGGAGTCCCATGATGAGATTGT |
|  | qBAX-F | CCCGAGAGGTCTTTTTCCGAG |
|  | qBAX-R | CCAGCCCATGATGGTTCTGAT |
|  | qNOXA-F | ACCAAGCCGGATTTGCGATT |
|  | qNOXA-R | ACTTGCACTTGTTCCTCGTGG |

**SUPPLEMENTAL FIGURE 1.** Validate constructions by PCR and Sanger sequences. (**A**) Clones were screened by PCR with pretarget-F/LHA-R which would generate 1452-bp, 2137-bp, and 2752-bp PCR products, respectively. (**B**) PCR products were further verified by Sanger sequencing. Both fragments were consistent with the expected theoretical sequences.


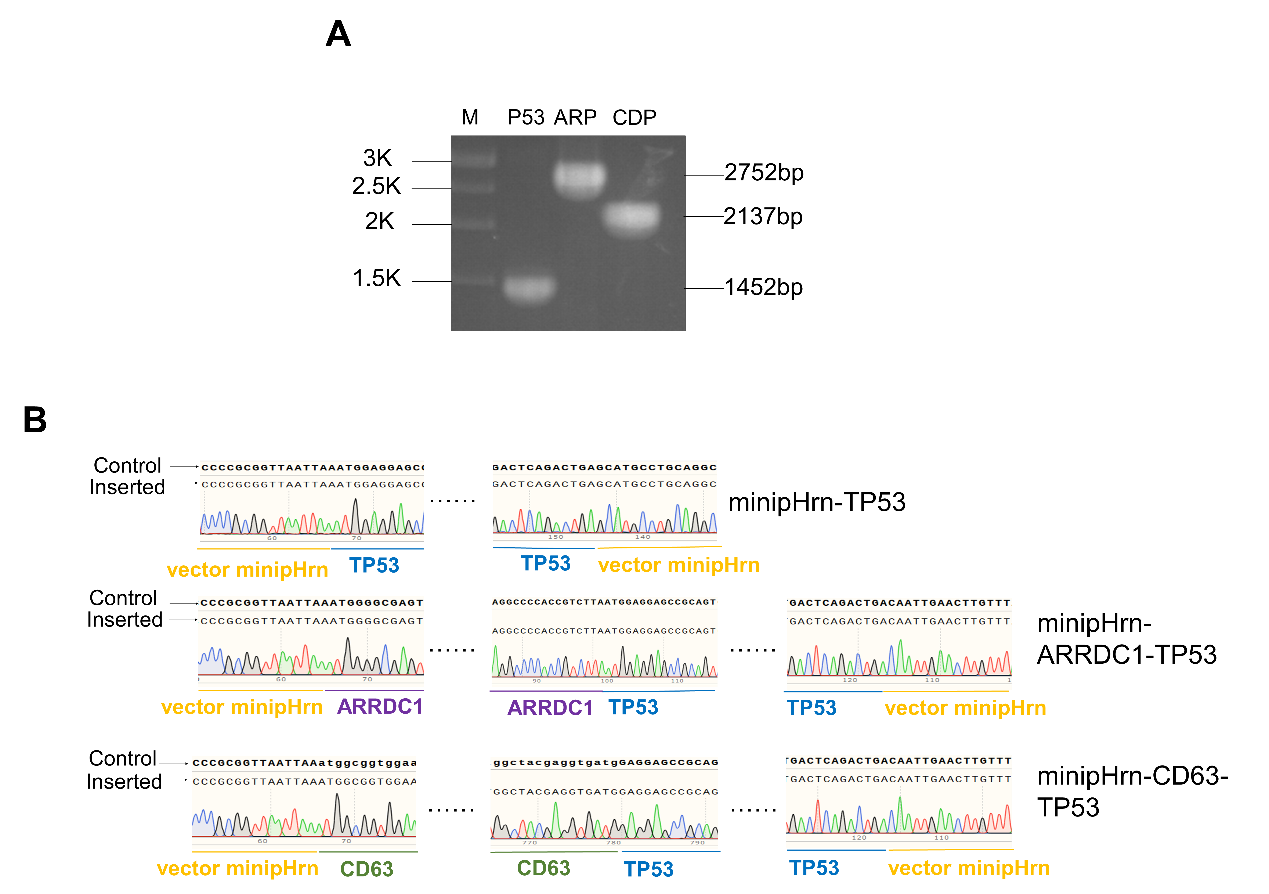


**SUPPLEMENTAL FIGURE 2.**  Diameter (median) of sEVs in nm measured by NTA (Data are mean ± SEM, n > 3 per group, ns, not significant).
