# Supplementary figures and images for: Fusion with ARRDC1 or CD63: A Strategy to Enhance p53 Loading into Extracellular Vesicles for Tumor Suppression
Source: Biomolecules. 2024 May 16;14(5):591. doi: 10.3390/biom14050591 (PMC11118238; doi:10.3390/biom14050591)

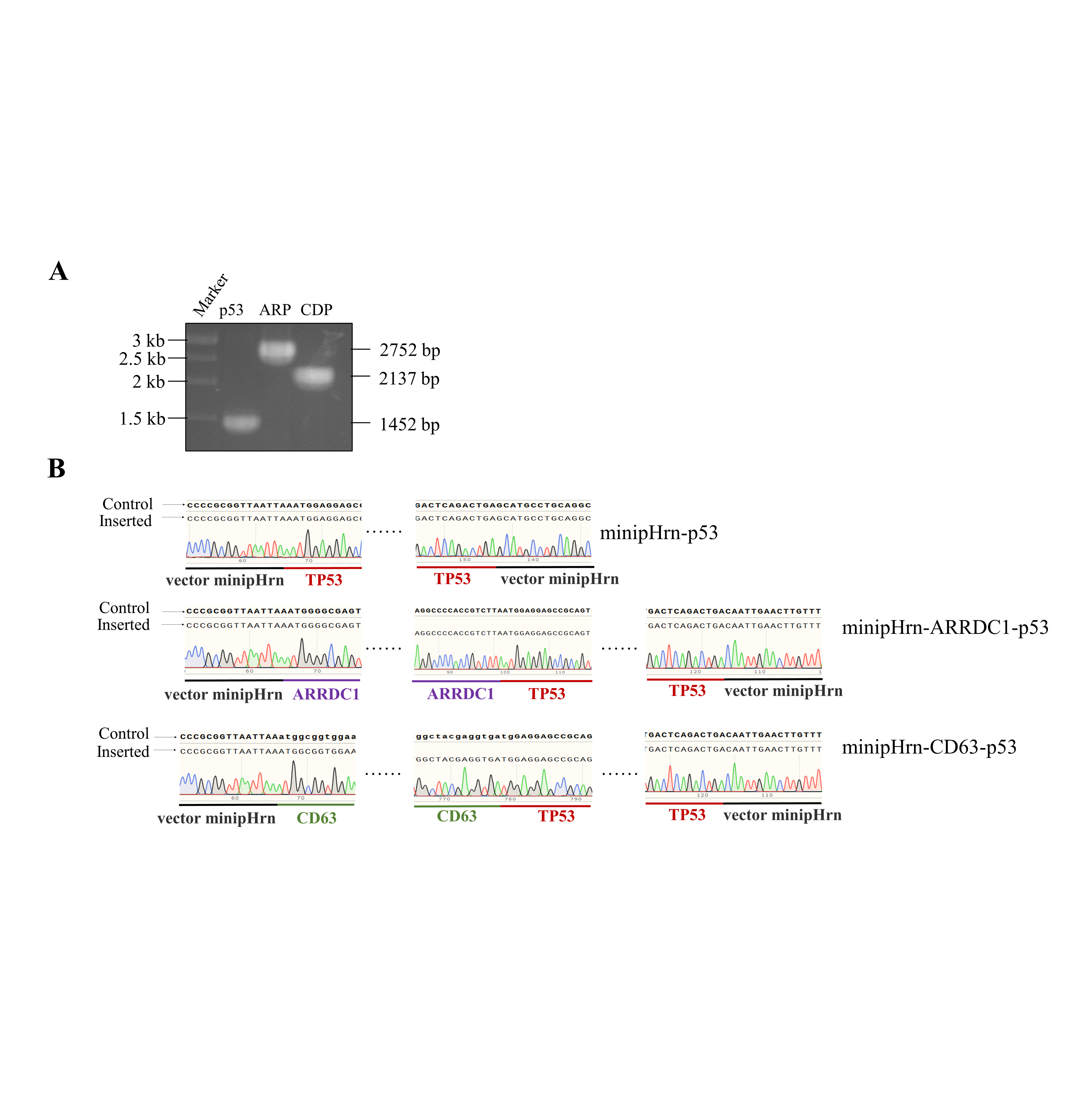

Supplement: Supplementary file 1 [file biomolecules-14-00591-s001.zip › Sup_ARP/sup figure 1.tif]

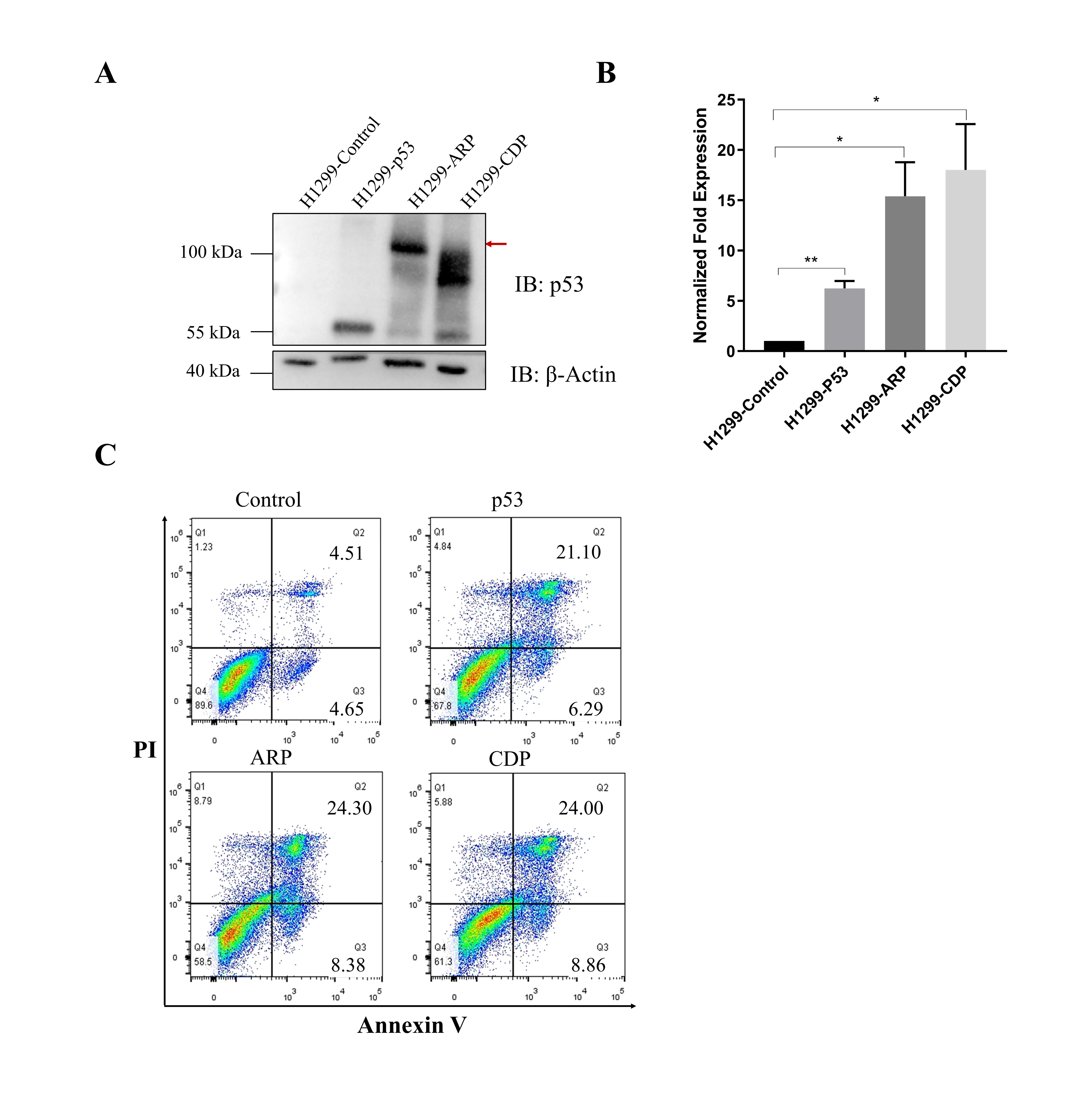

Supplement: Supplementary file 1 [file biomolecules-14-00591-s001.zip › Sup_ARP/sup figure 2.tif]

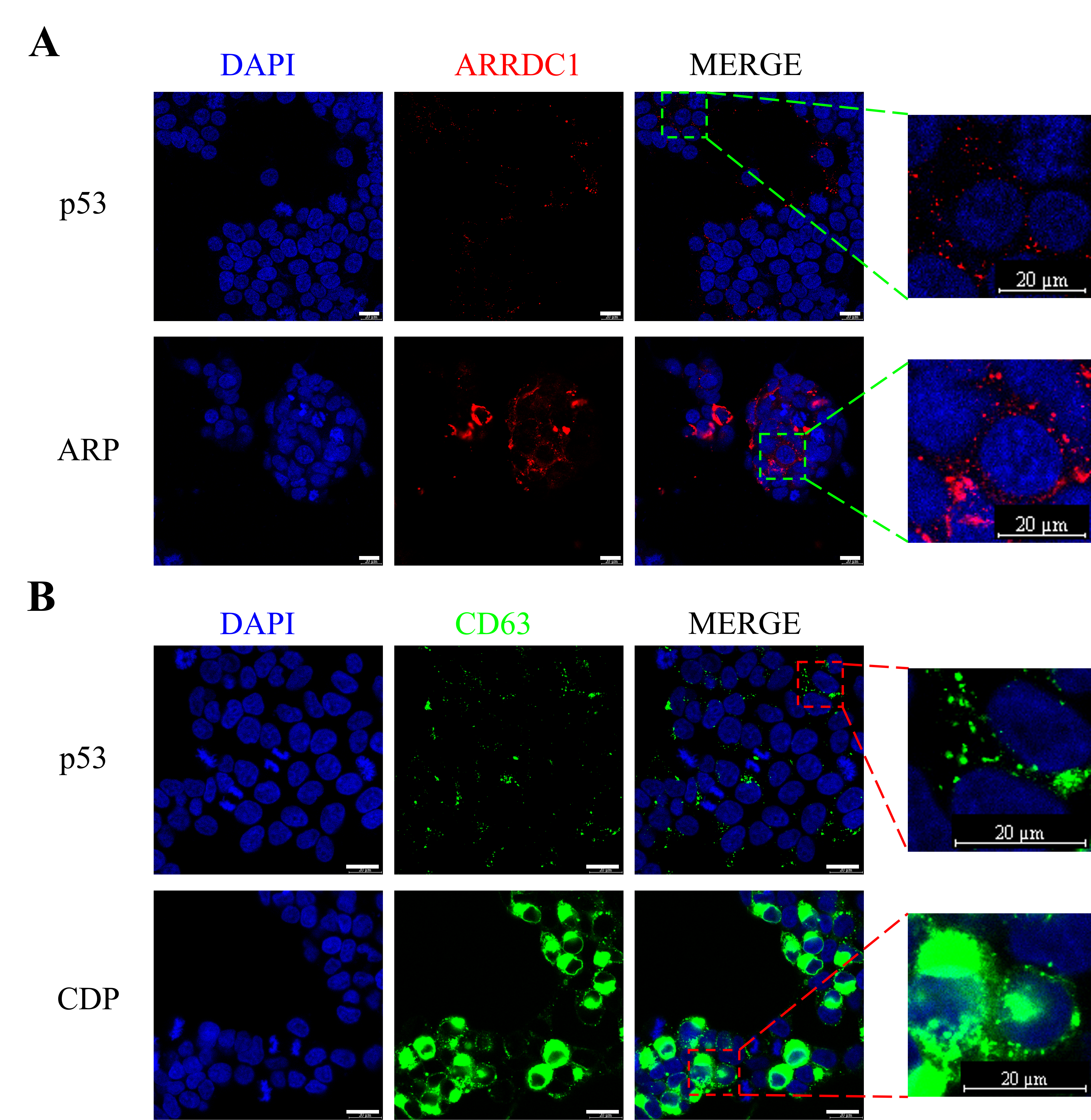

Supplement: Supplementary file 1 [file biomolecules-14-00591-s001.zip › Sup_ARP/sup figure 3_R.tif]

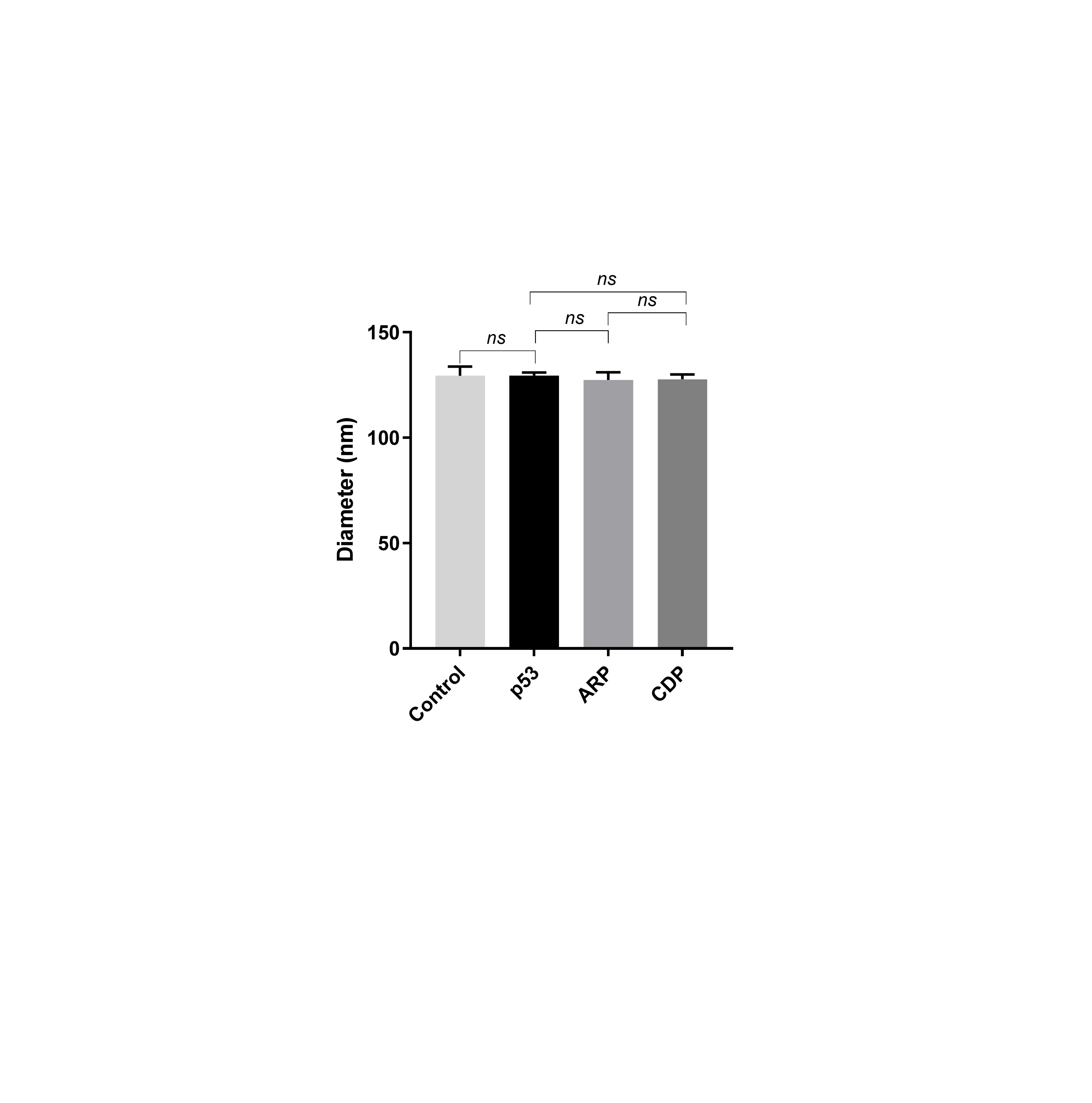

Supplement: Supplementary file 1 [file biomolecules-14-00591-s001.zip › Sup_ARP/sup figure 4.tif]

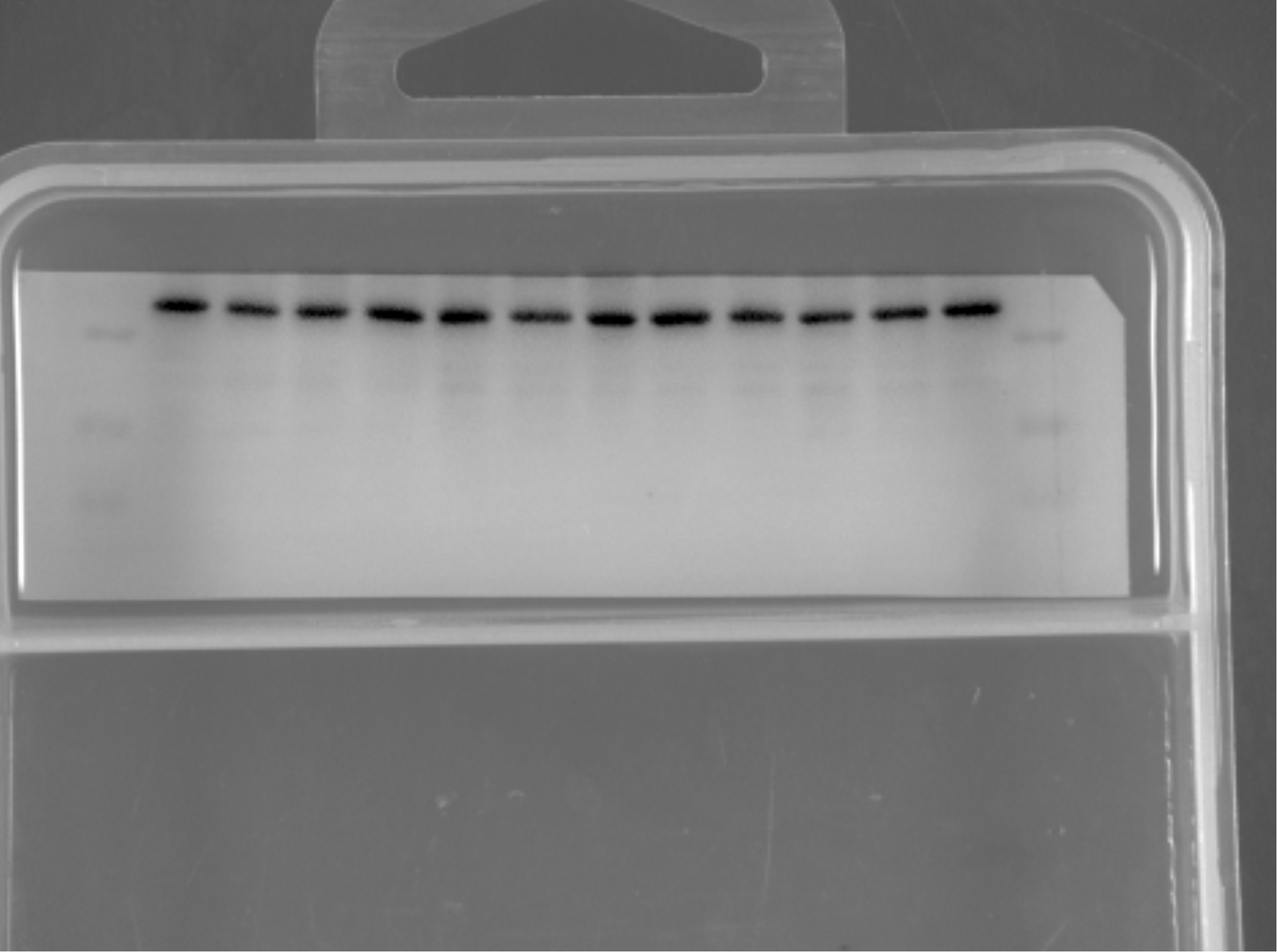

Supplement: Supplementary file 1 [file biomolecules-14-00591-s001.zip › WB_pictures_original/293T_CELL/ACTIN-1.tif]

## Slide 1
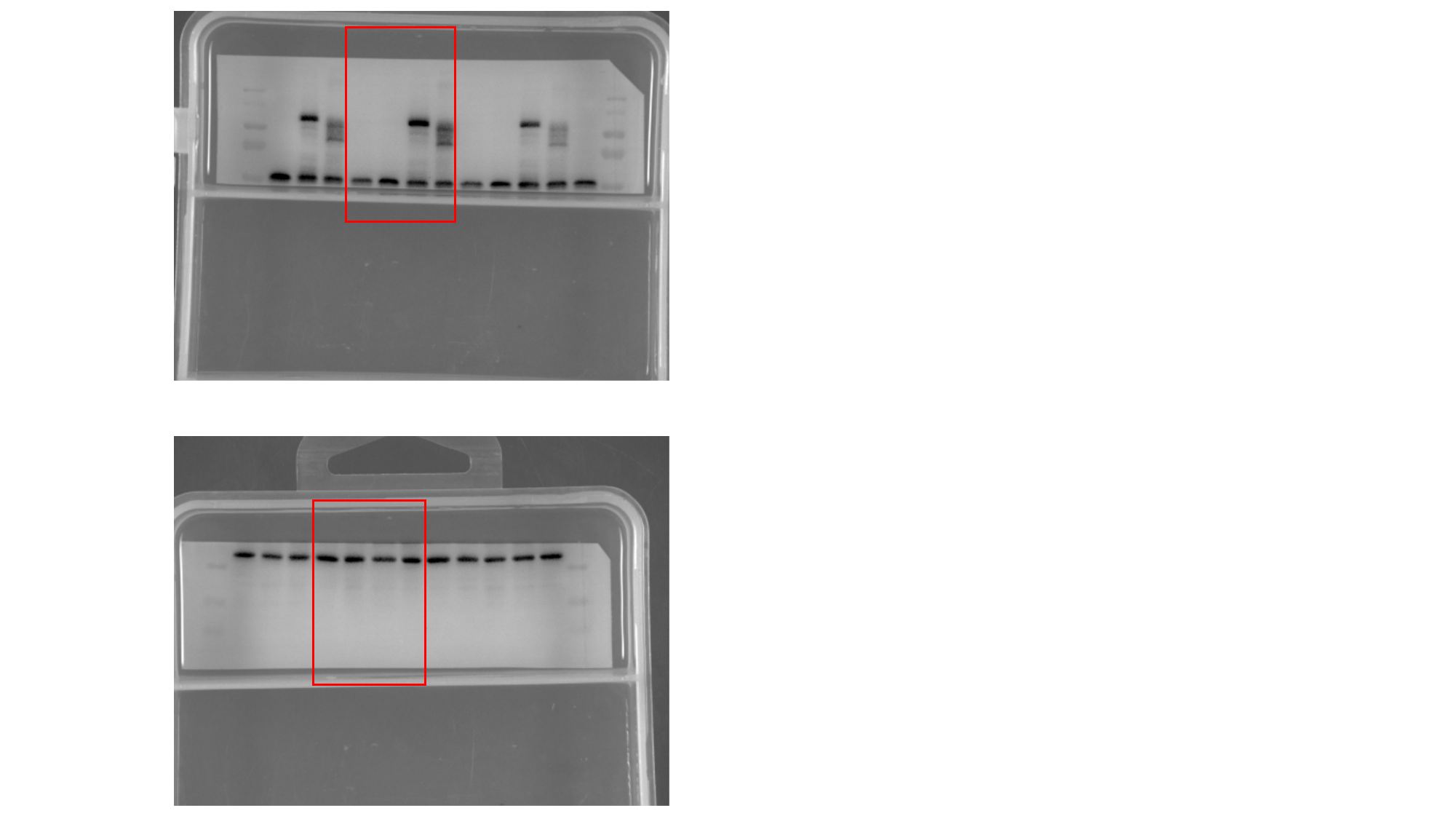

Supplement: Supplementary file 1 [file biomolecules-14-00591-s001.zip › WB_pictures_original/293T_CELL/Details_293T_CELL.pptx]

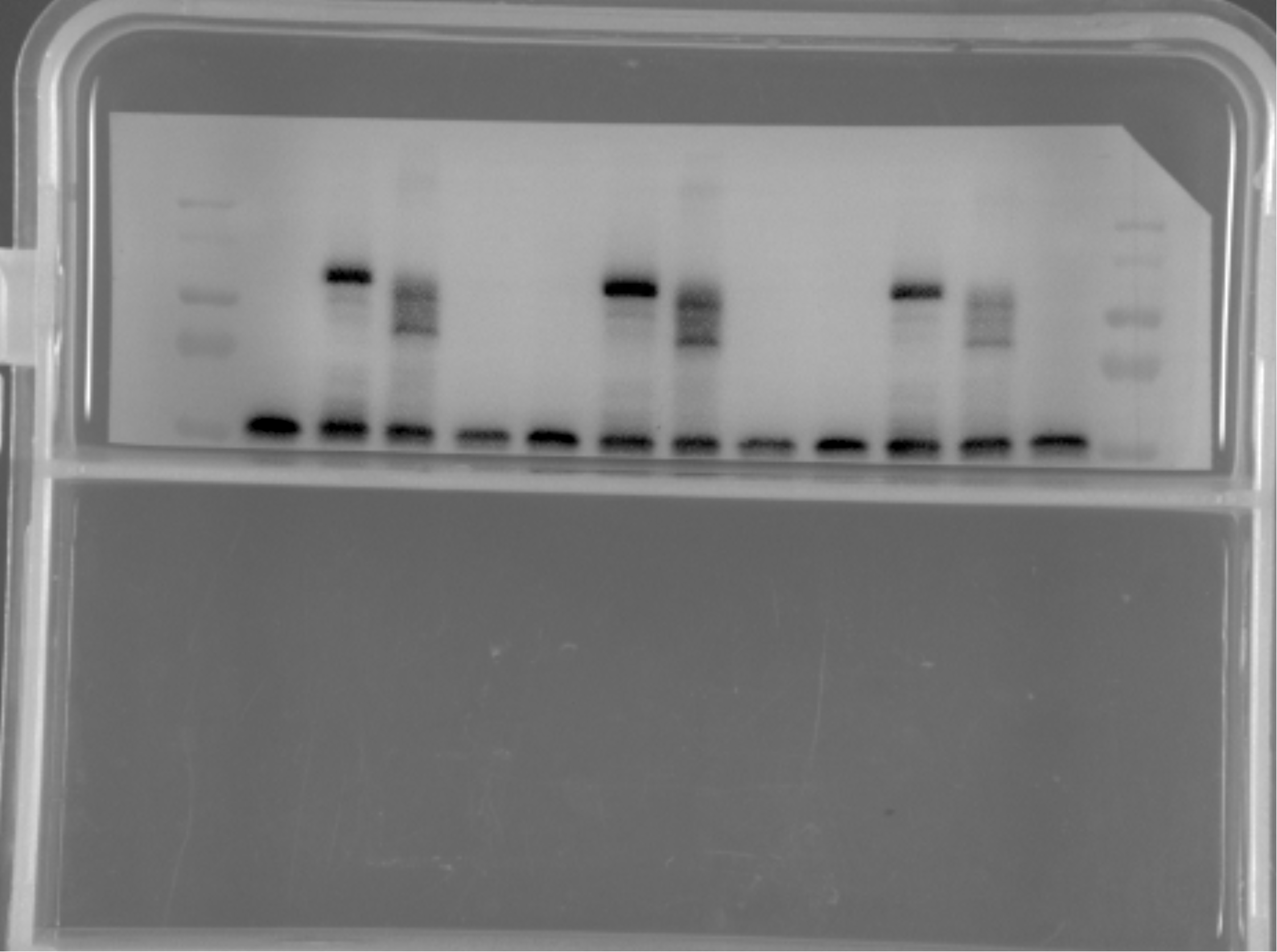

Supplement: Supplementary file 1 [file biomolecules-14-00591-s001.zip › WB_pictures_original/293T_CELL/P53-1.tif]

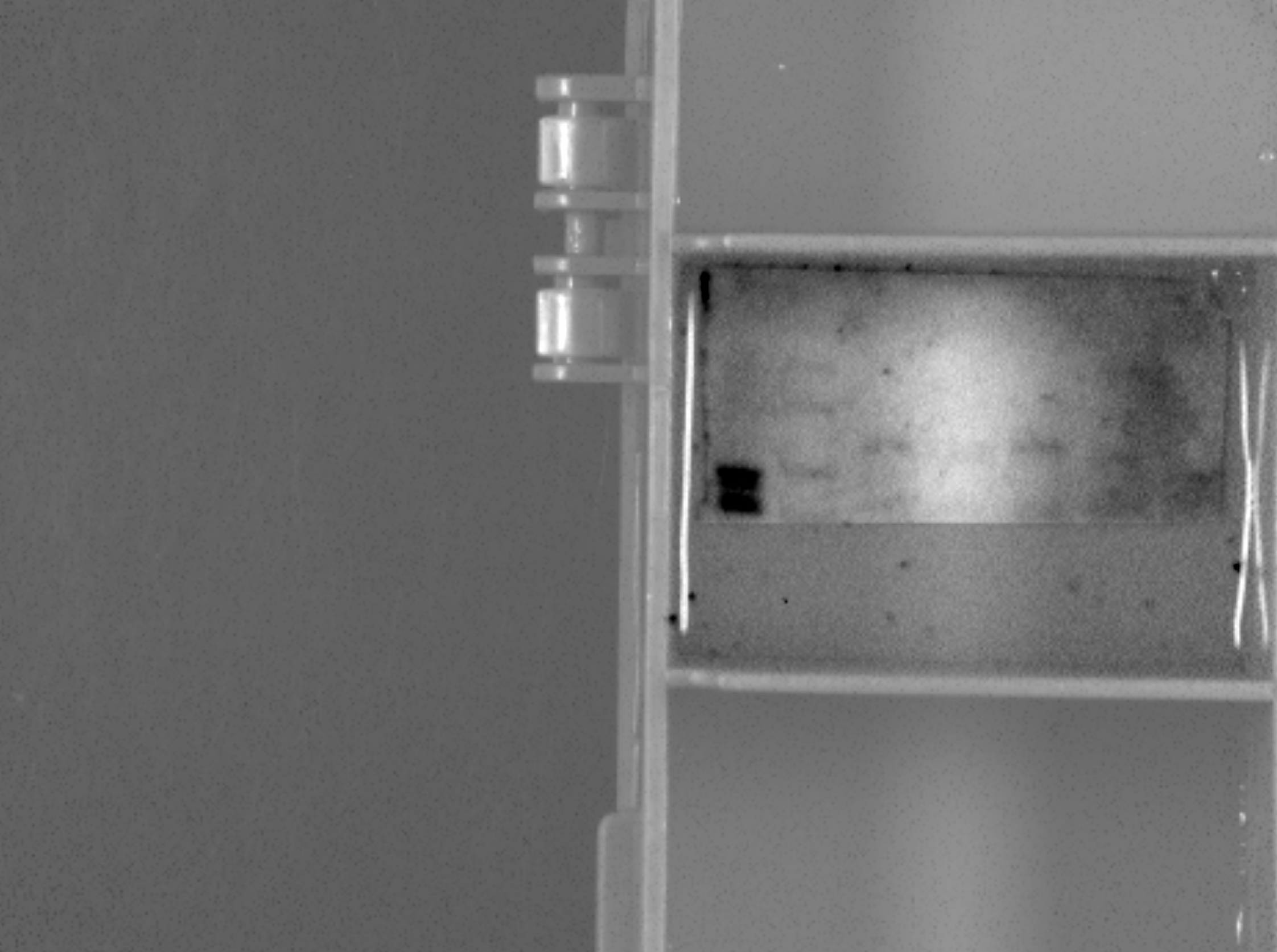

Supplement: Supplementary file 1 [file biomolecules-14-00591-s001.zip › WB_pictures_original/293T_EV_MARKERS/Calnexin-Administrator 2023-06-07_11h23m33s_Exposure_12.8sec+293-cal-02-m.tif]

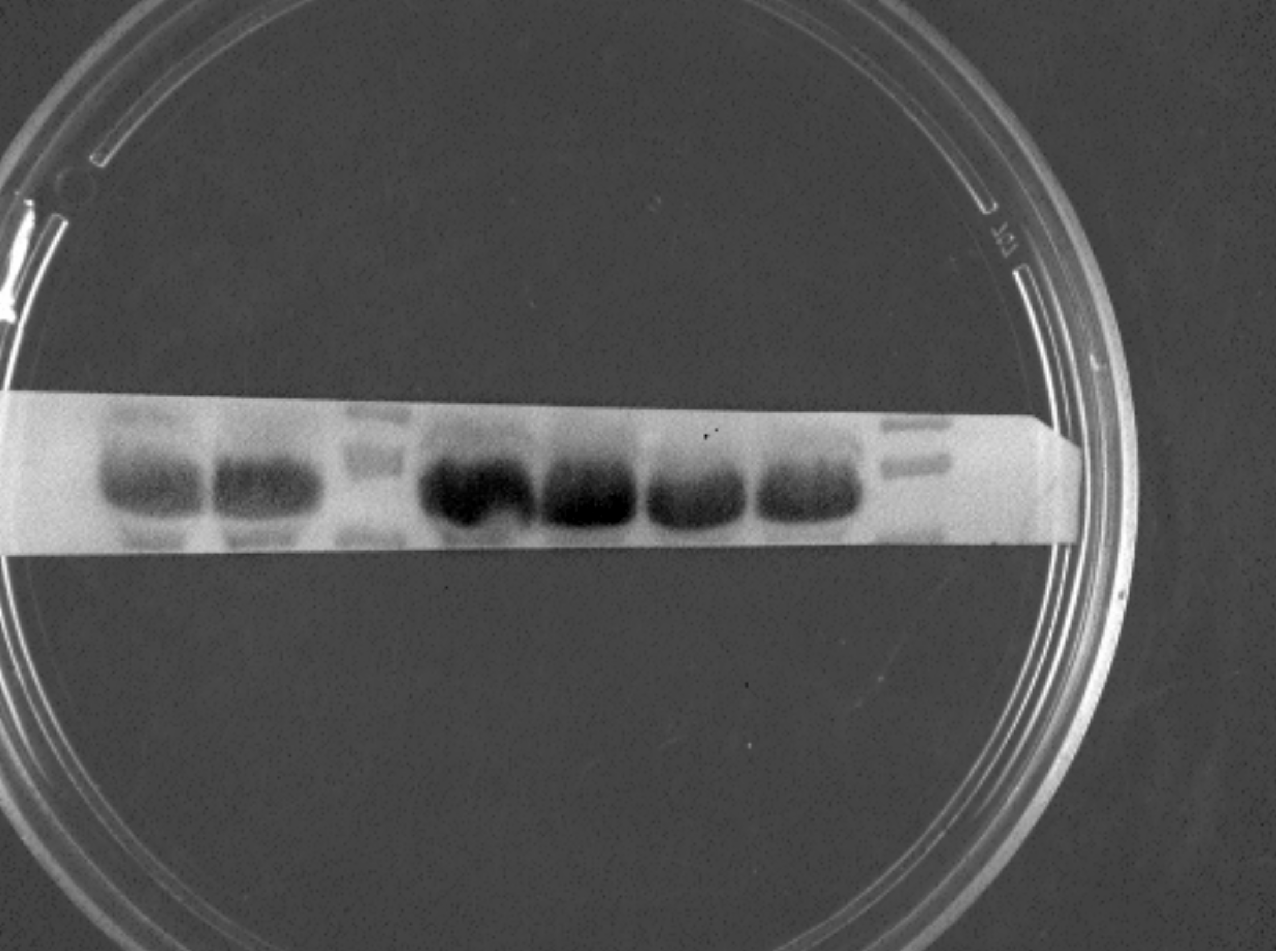

Supplement: Supplementary file 1 [file biomolecules-14-00591-s001.zip › WB_pictures_original/293T_EV_MARKERS/CD63_Exposure_34.1sec.tif]

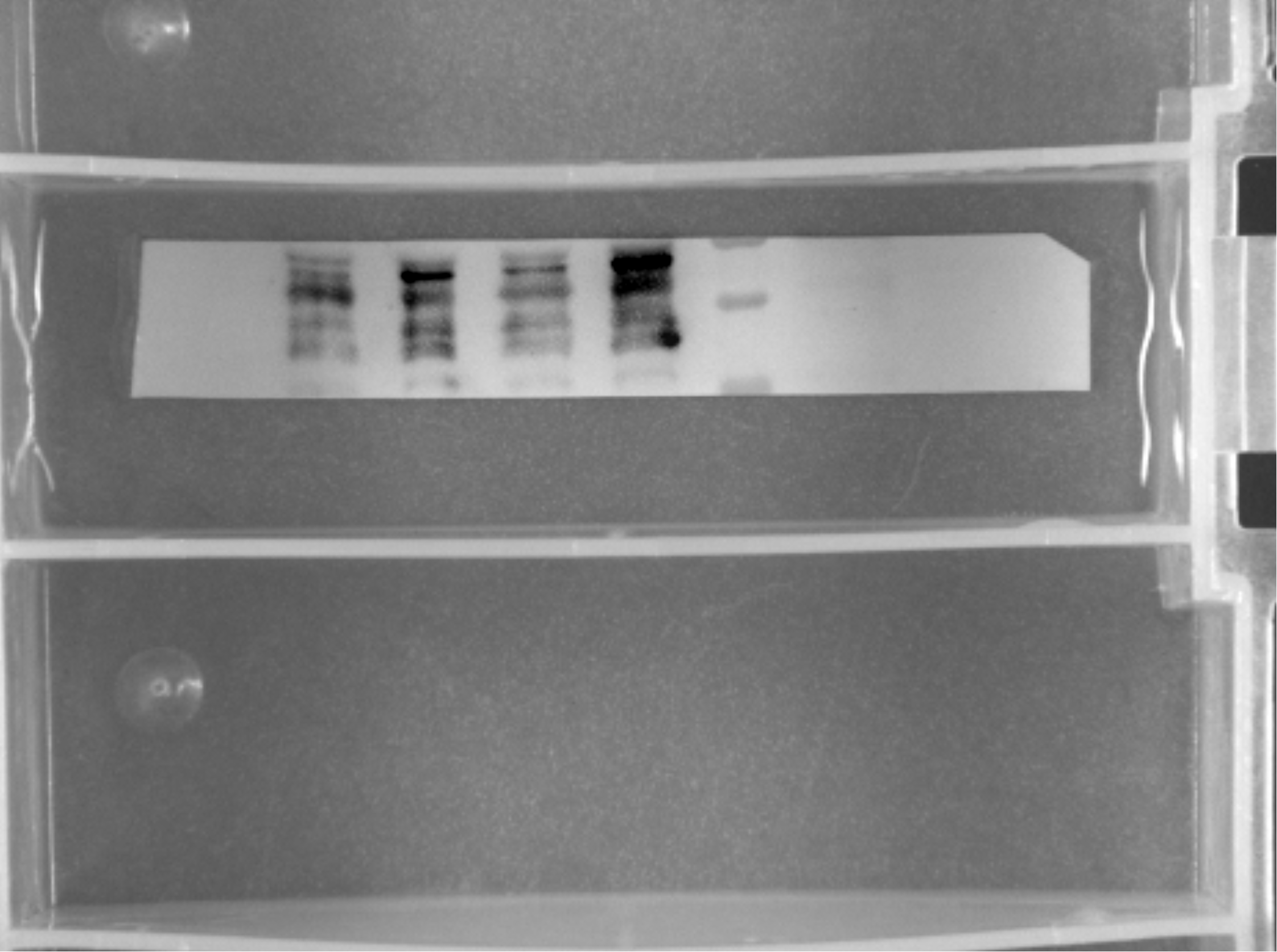

Supplement: Supplementary file 1 [file biomolecules-14-00591-s001.zip › WB_pictures_original/293T_EV_MARKERS/CD9_3_ladder.tif]

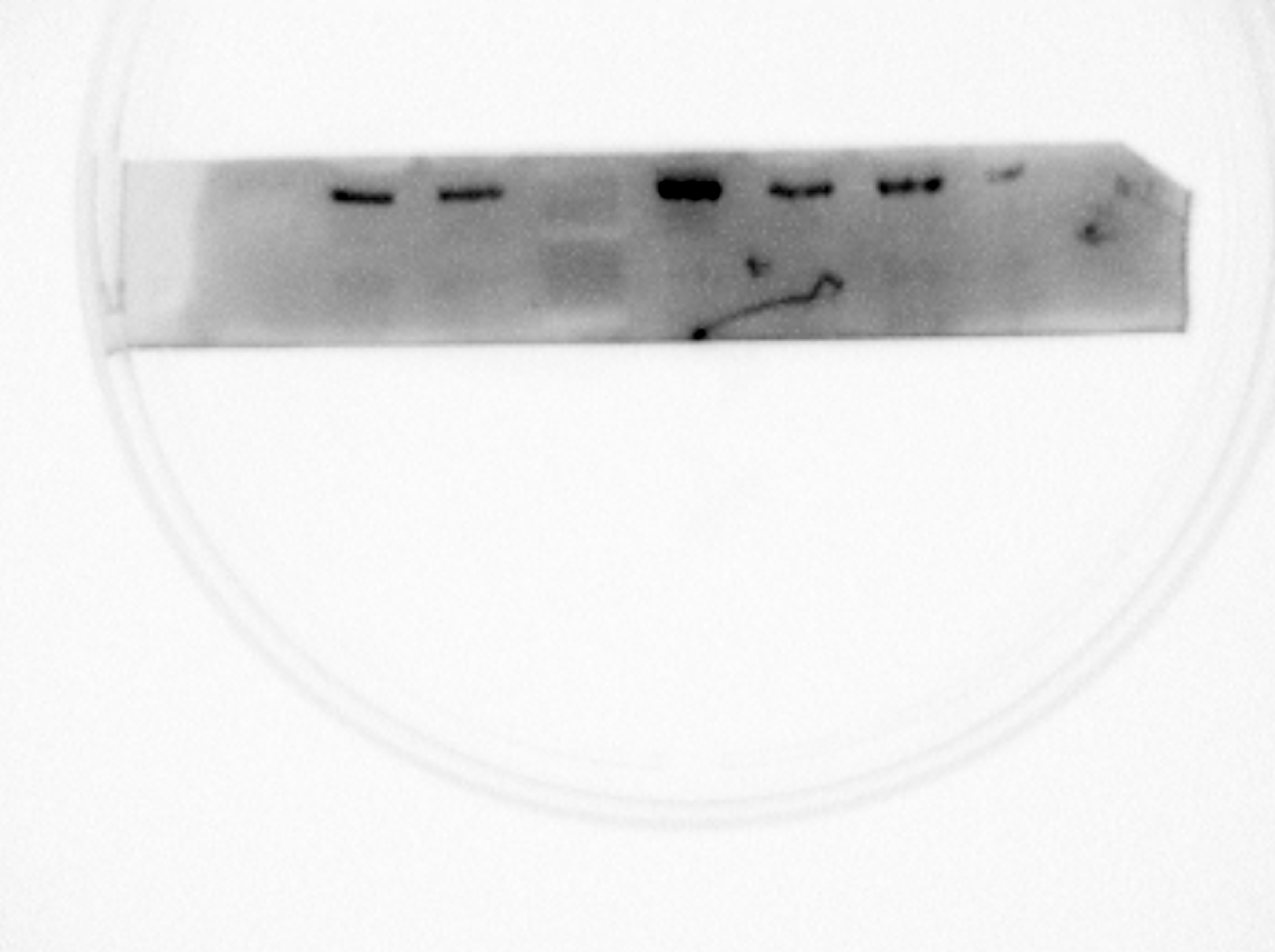

Supplement: Supplementary file 1 [file biomolecules-14-00591-s001.zip › WB_pictures_original/293T_EV_MARKERS/TSG101_1.tif]

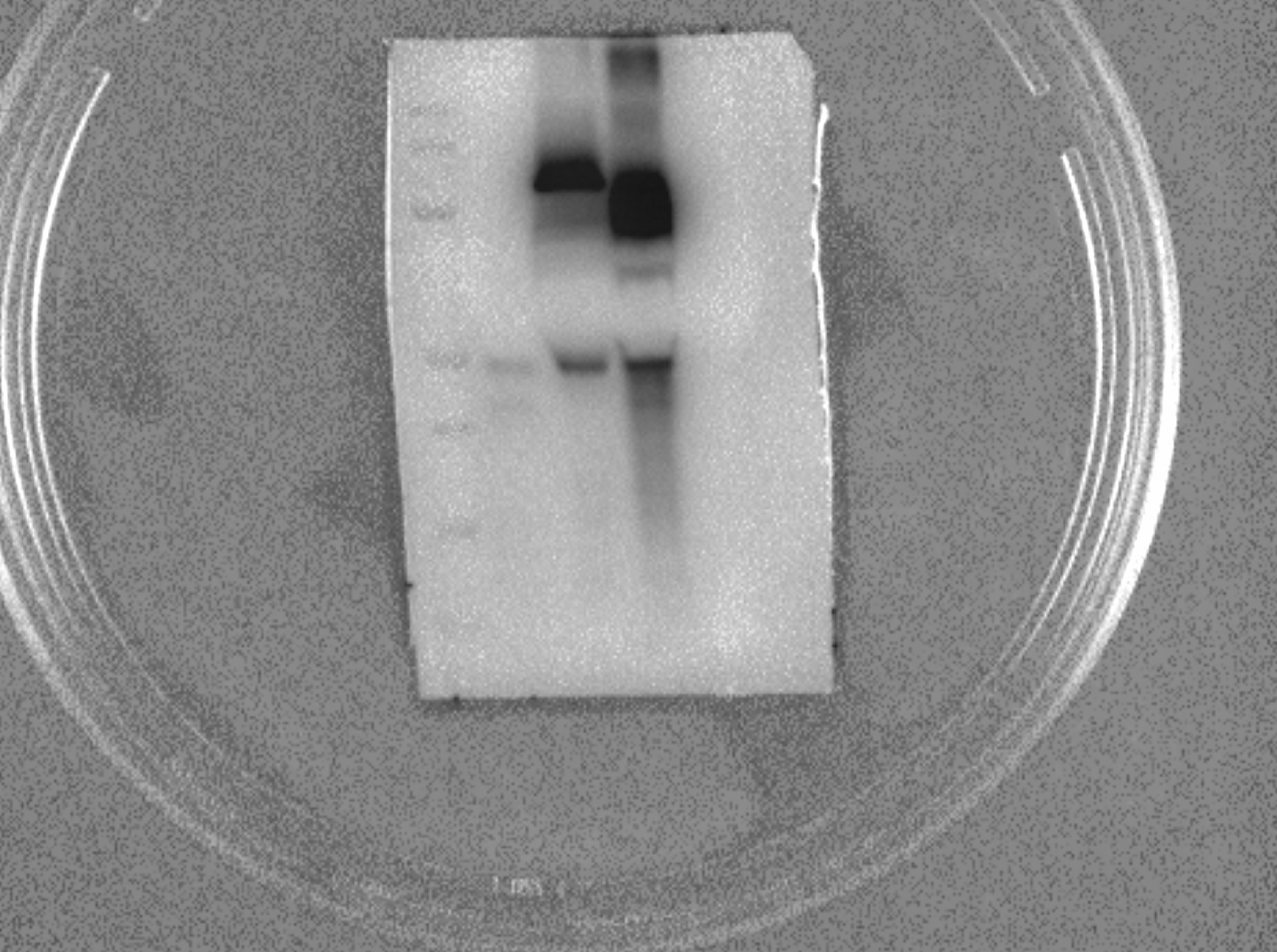

Supplement: Supplementary file 1 [file biomolecules-14-00591-s001.zip › WB_pictures_original/293T_EV_P53/293E-p53_marker+293E-p53_29.3sec.tif]

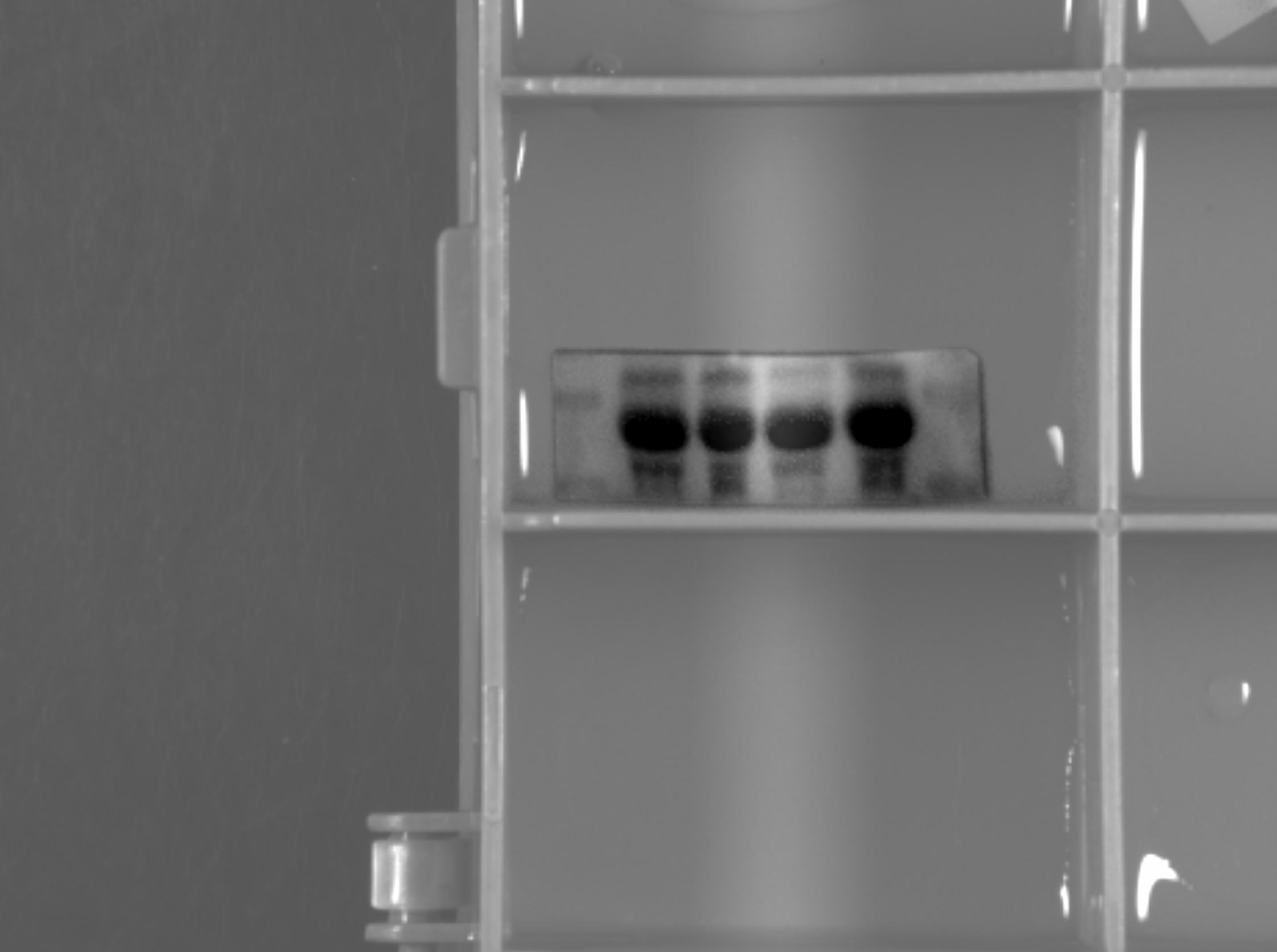

Supplement: Supplementary file 1 [file biomolecules-14-00591-s001.zip › WB_pictures_original/293T_EV_P53/cd63-05-m+cd63-05.tif]

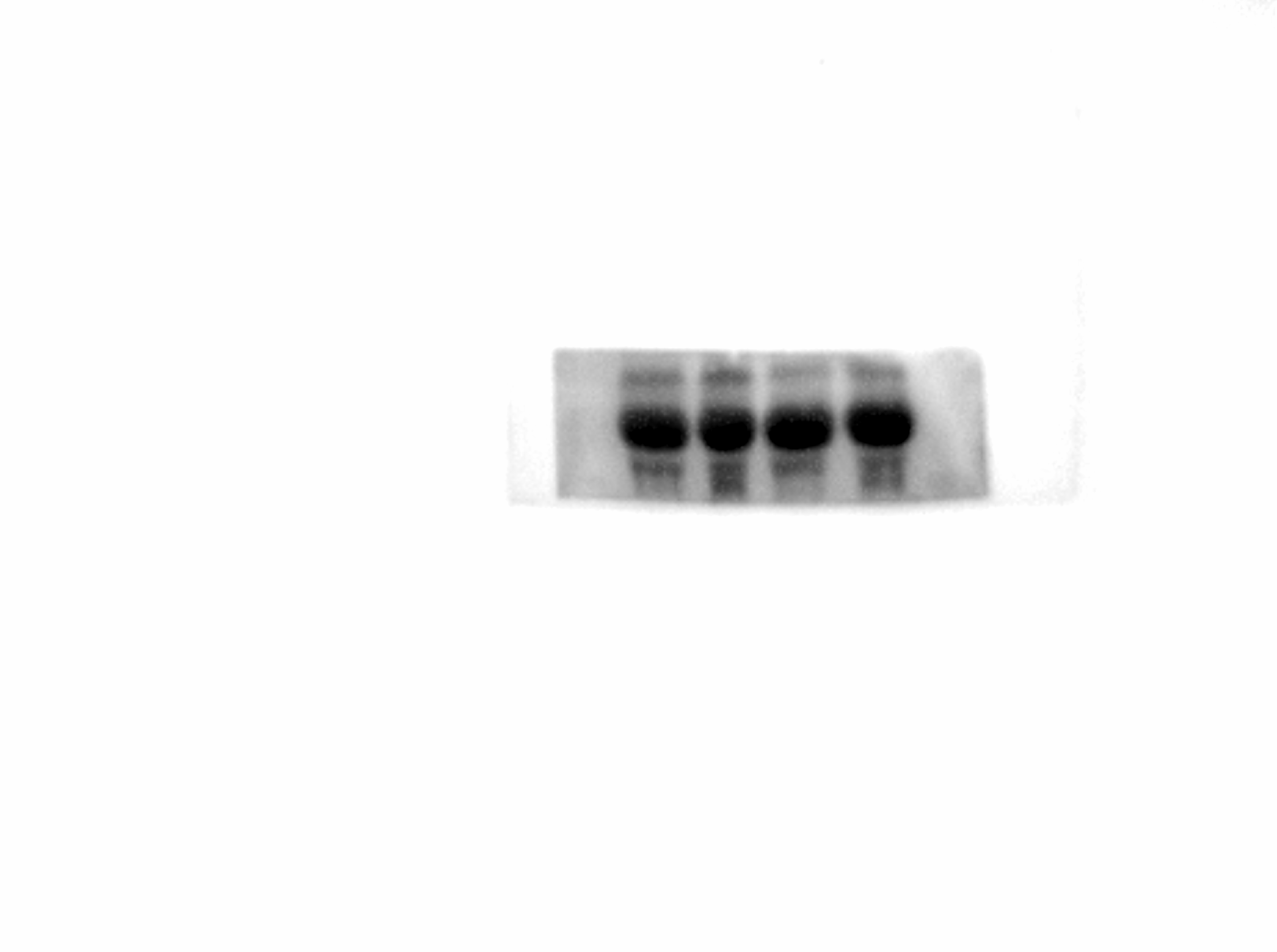

Supplement: Supplementary file 1 [file biomolecules-14-00591-s001.zip › WB_pictures_original/293T_EV_P53/cd63-05.tif]

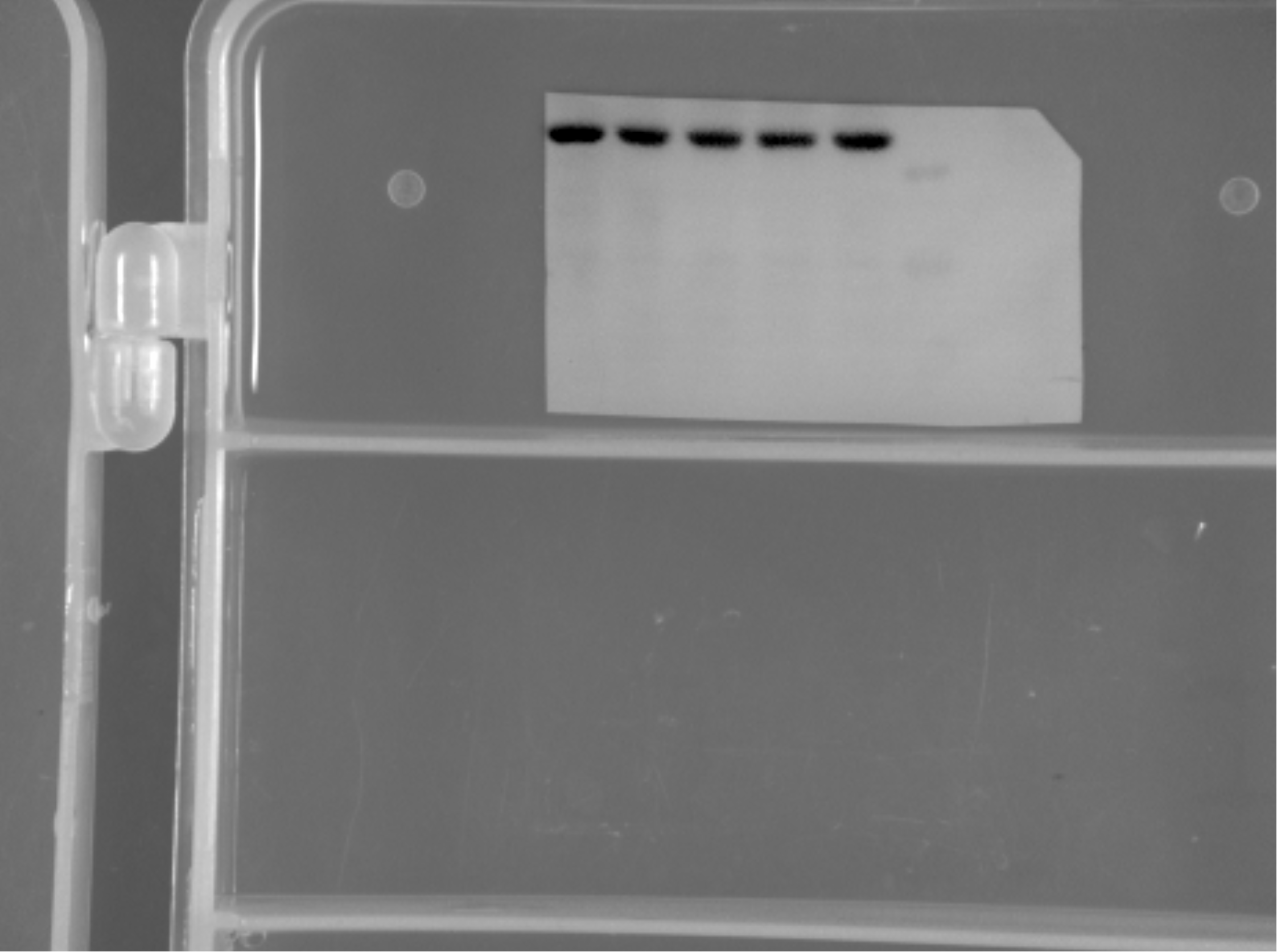

Supplement: Supplementary file 1 [file biomolecules-14-00591-s001.zip › WB_pictures_original/H1299+EV_P53/ACTIN-3.tif]

## Slide 1
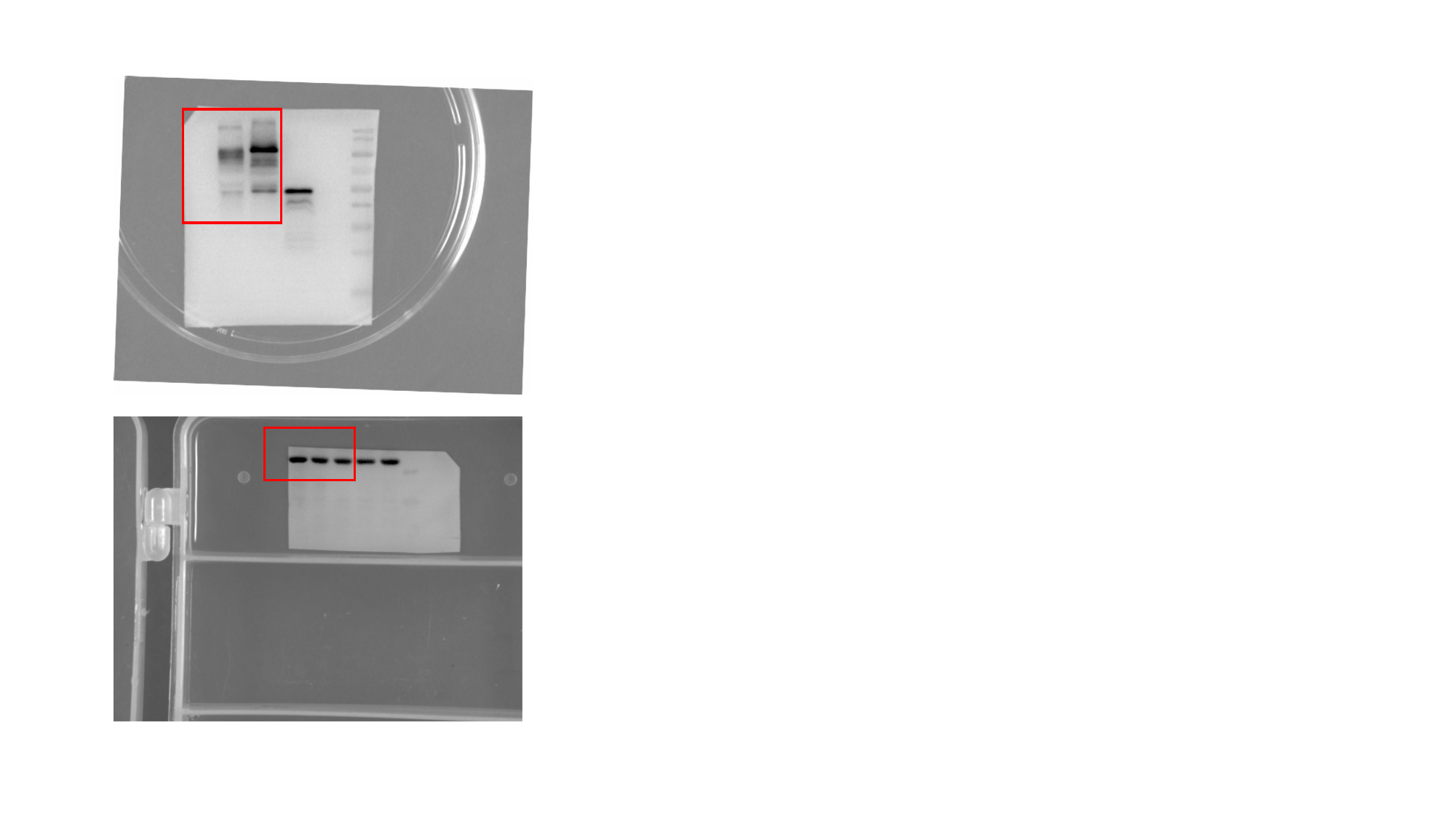

Supplement: Supplementary file 1 [file biomolecules-14-00591-s001.zip › WB_pictures_original/H1299+EV_P53/Details_H1299+E.pptx]

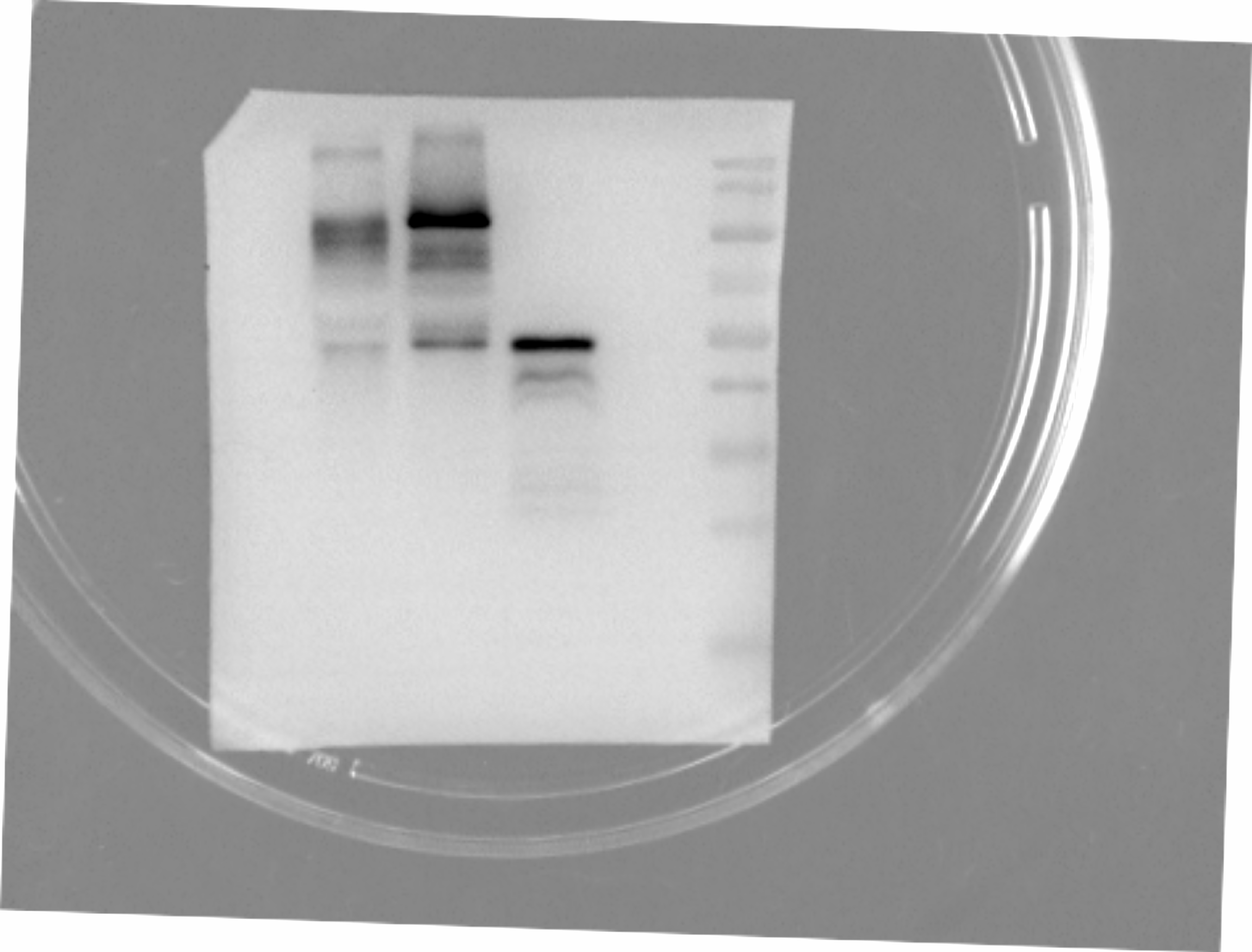

Supplement: Supplementary file 1 [file biomolecules-14-00591-s001.zip › WB_pictures_original/H1299+EV_P53/h12+E-p_19.5sec+h12-E-p-marker-2.tif]

## Slide 1
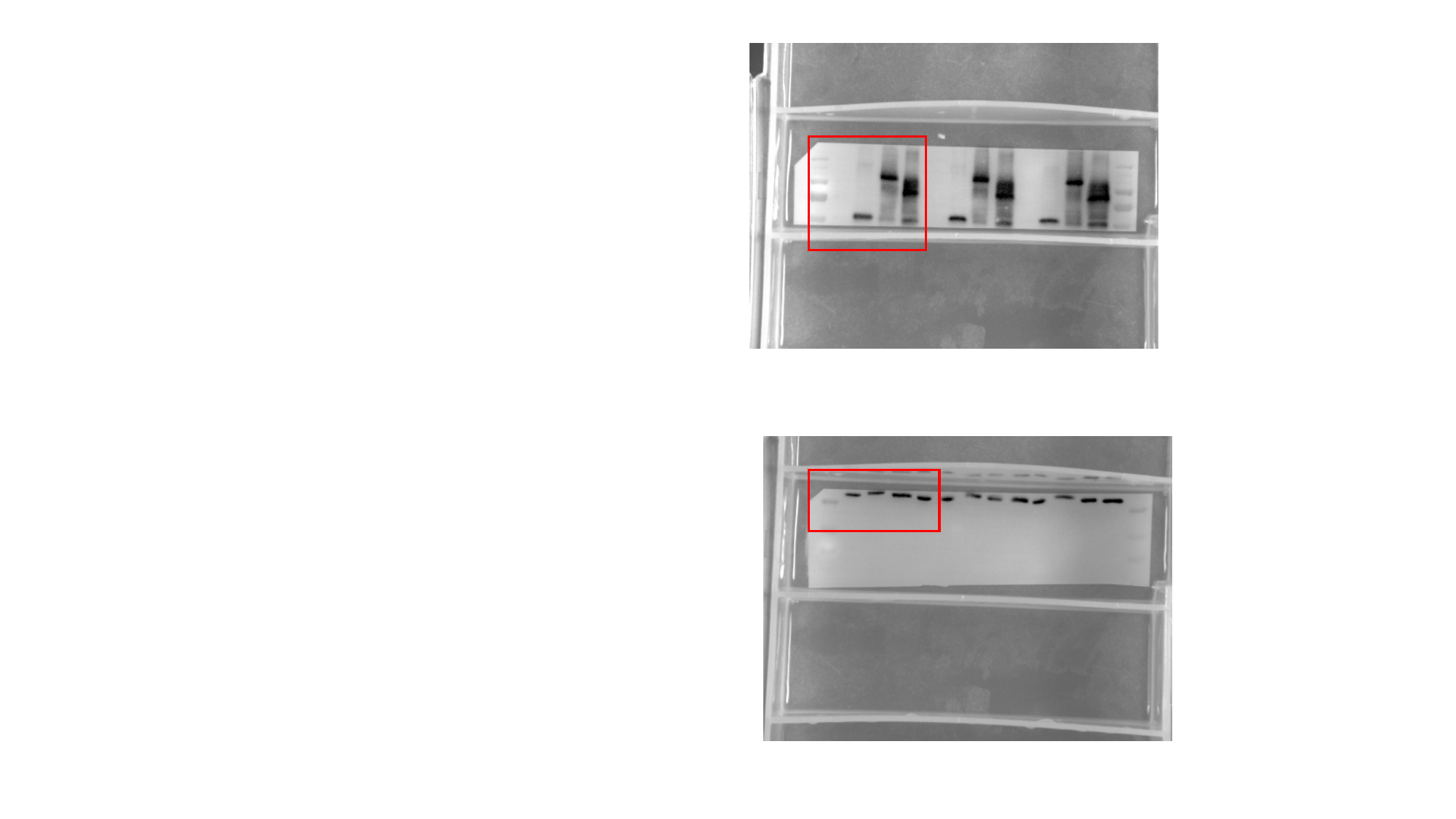

Supplement: Supplementary file 1 [file biomolecules-14-00591-s001.zip › WB_pictures_original/H1299_CELL/Details_H1299_CELL.pptx]

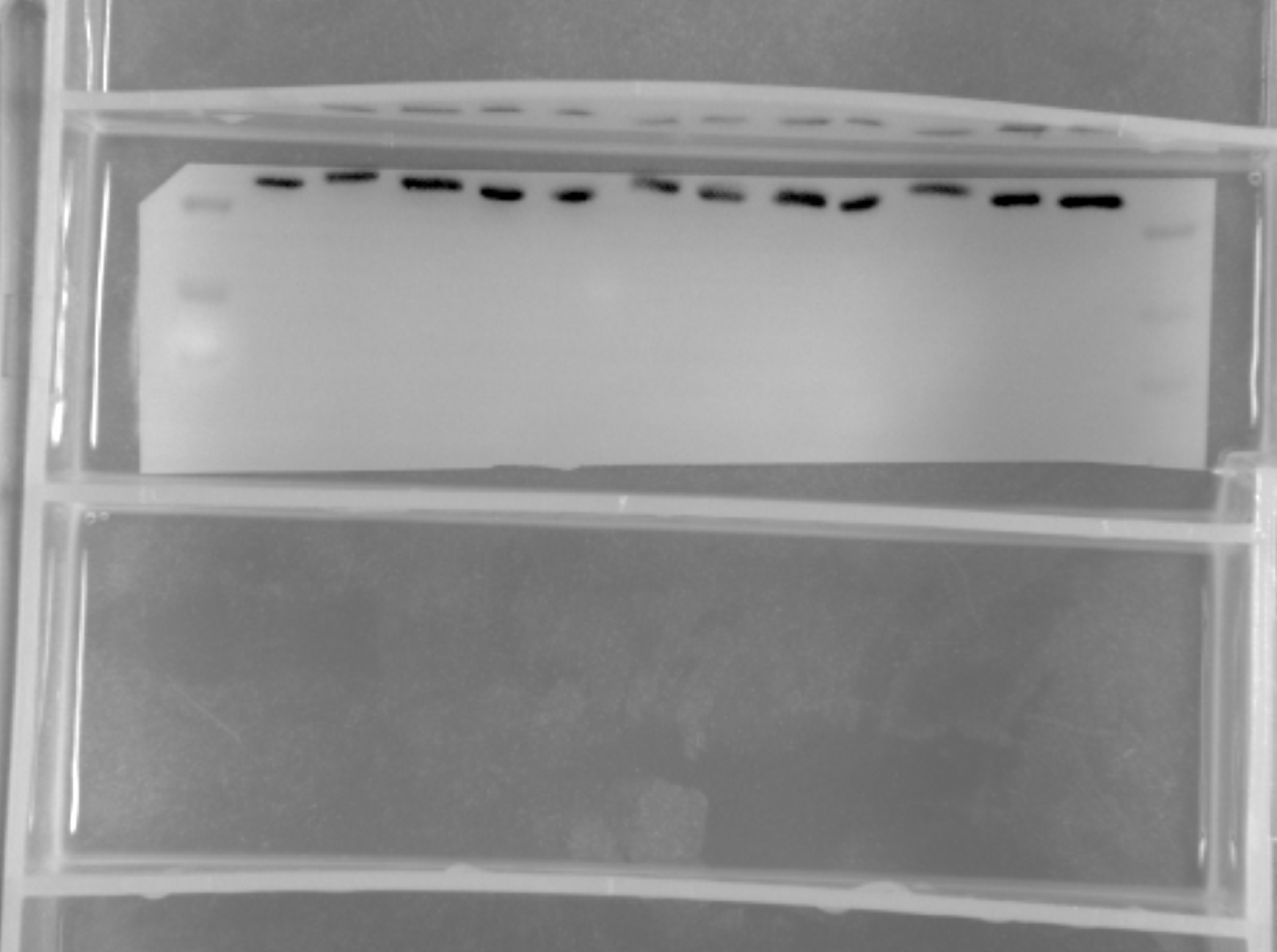

Supplement: Supplementary file 1 [file biomolecules-14-00591-s001.zip › WB_pictures_original/H1299_CELL/h1299-cell-actin-01+h1299-cell-actin-01-m-01.tif]

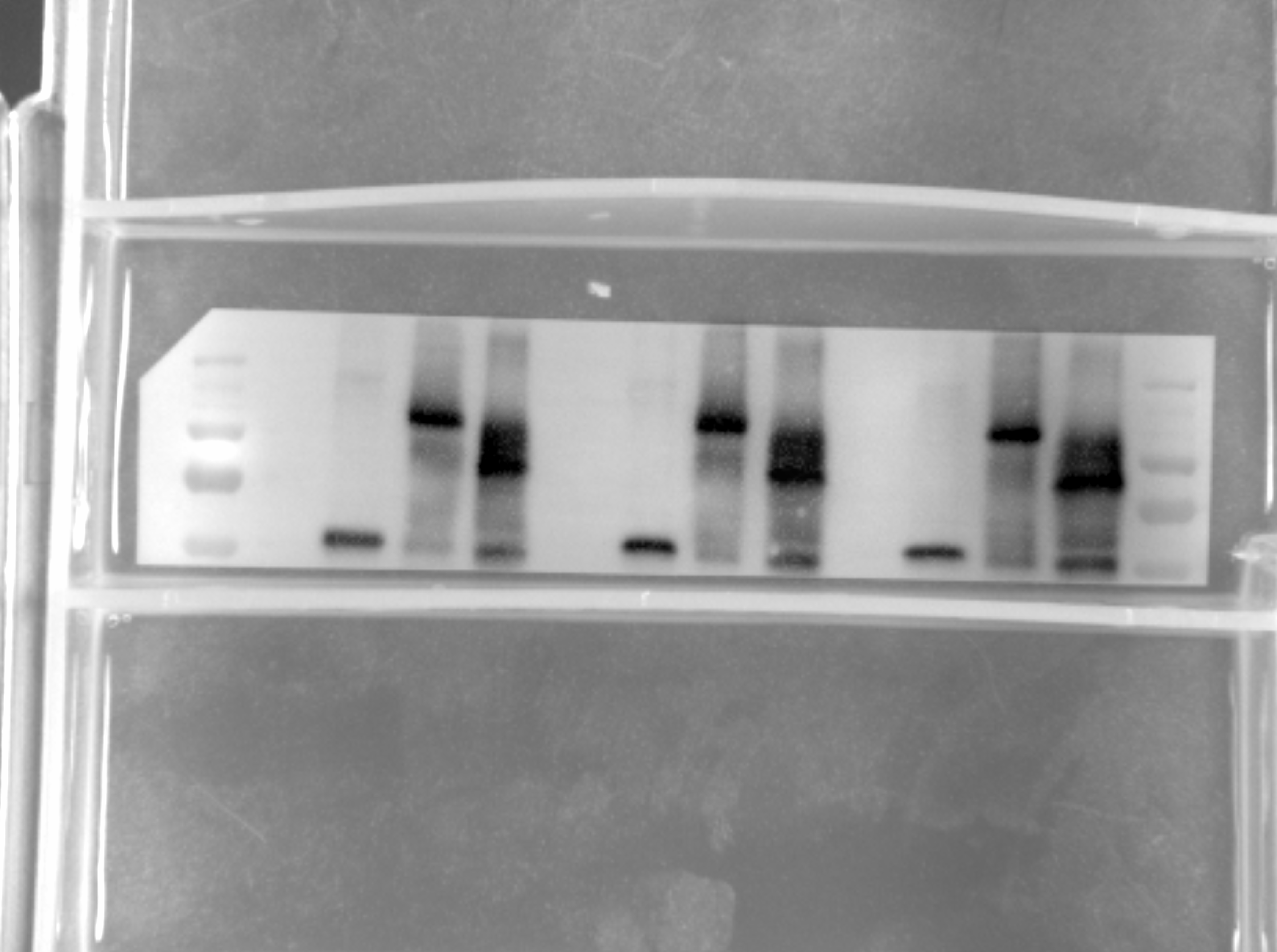

Supplement: Supplementary file 1 [file biomolecules-14-00591-s001.zip › WB_pictures_original/H1299_CELL/h1299-cell-p53-m+h1299-cell-p53-01-01.tif]

## Slide 1
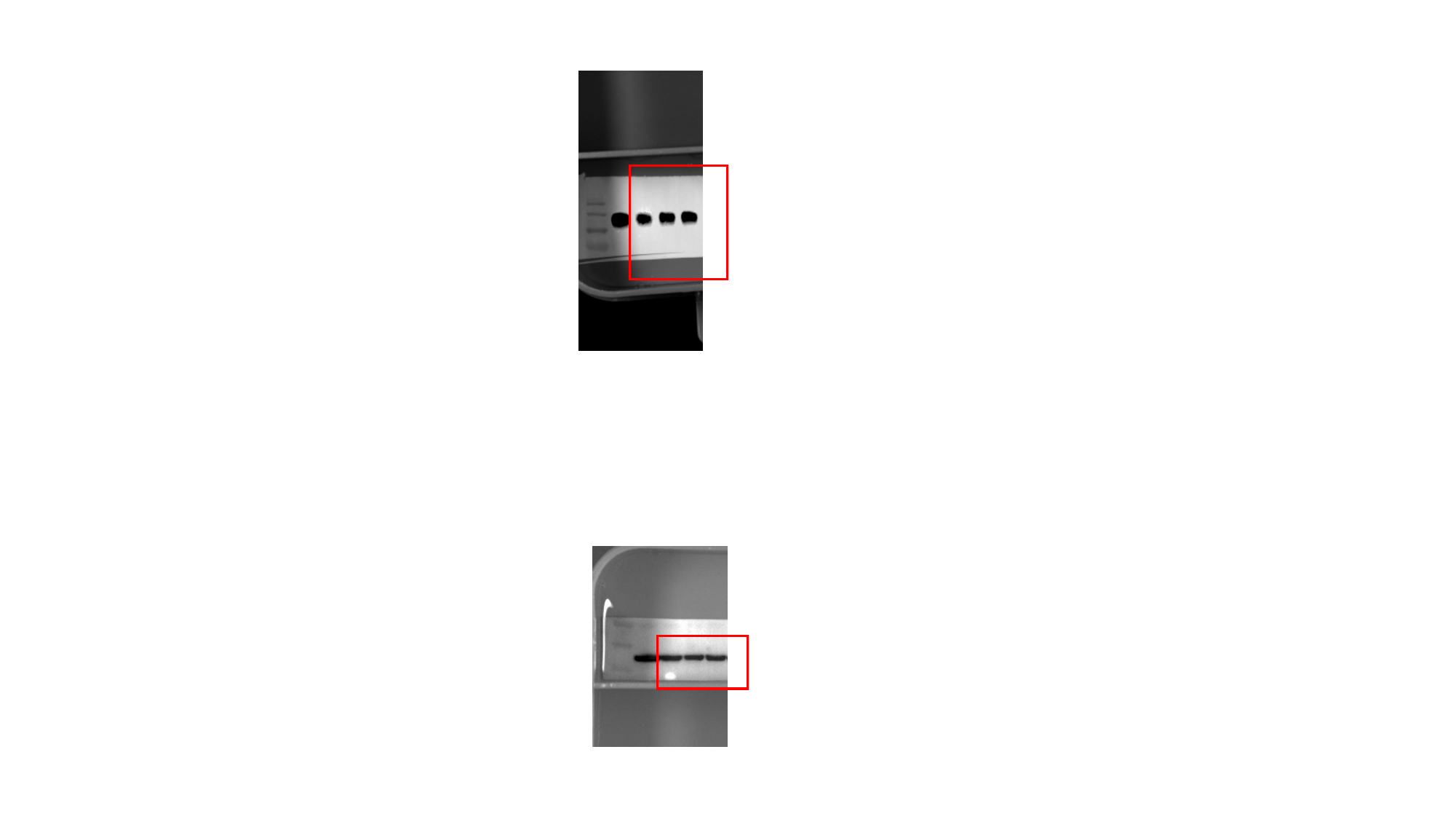

Supplement: Supplementary file 1 [file biomolecules-14-00591-s001.zip › WB_pictures_original/LAMP1/Details_LAMP1.pptx]

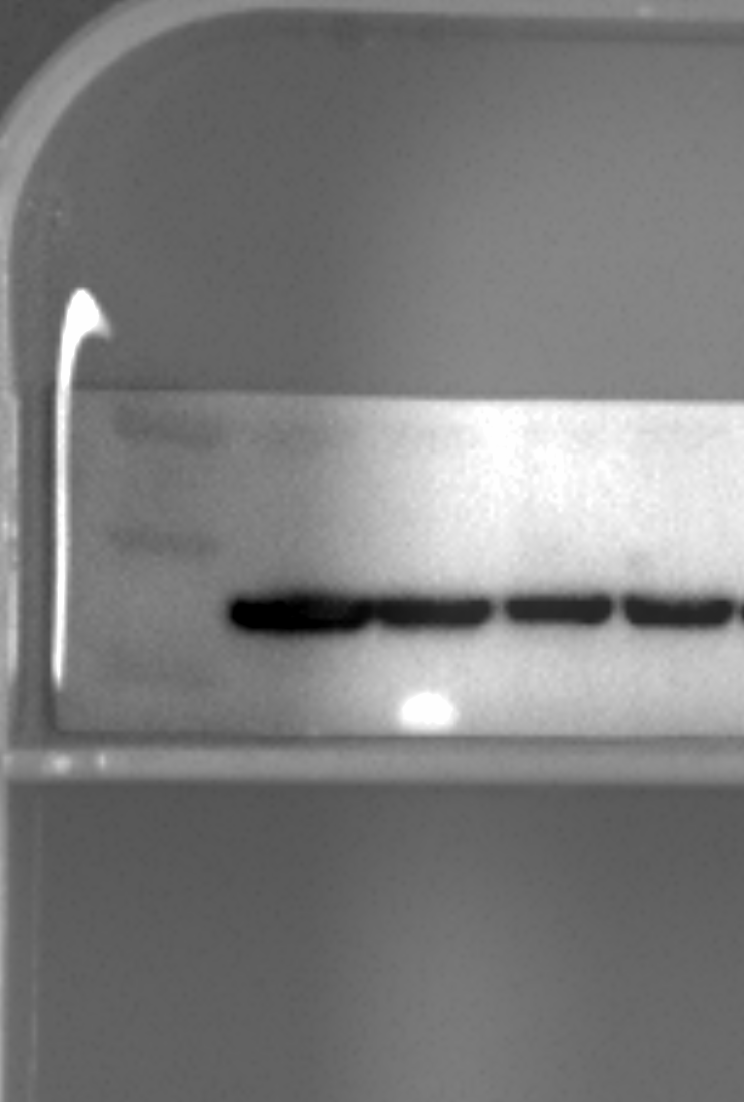

Supplement: Supplementary file 1 [file biomolecules-14-00591-s001.zip › WB_pictures_original/LAMP1/GAPDH-01-03-04-M+GAPDH-03-used.tif]

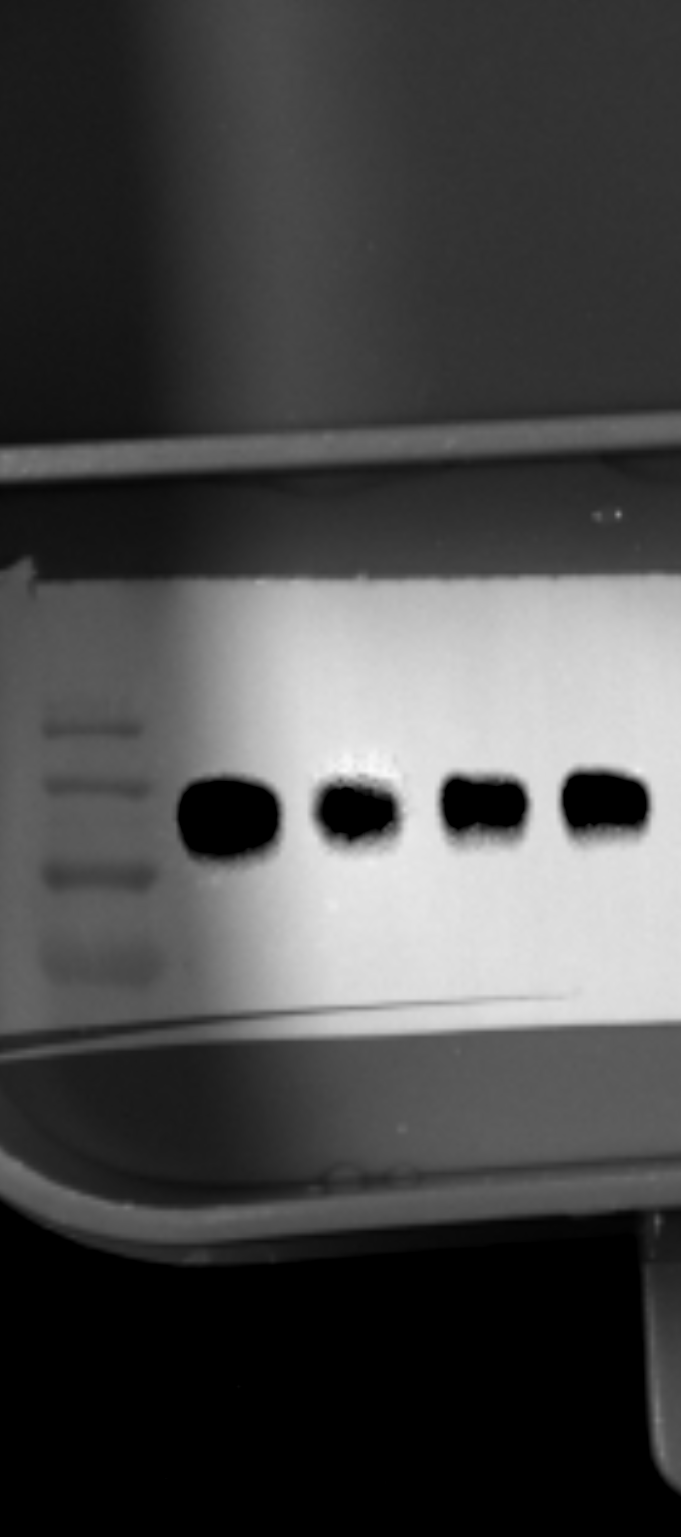

Supplement: Supplementary file 1 [file biomolecules-14-00591-s001.zip › WB_pictures_original/LAMP1/LAMP1-01-M+LAMP1-01-used.tif]

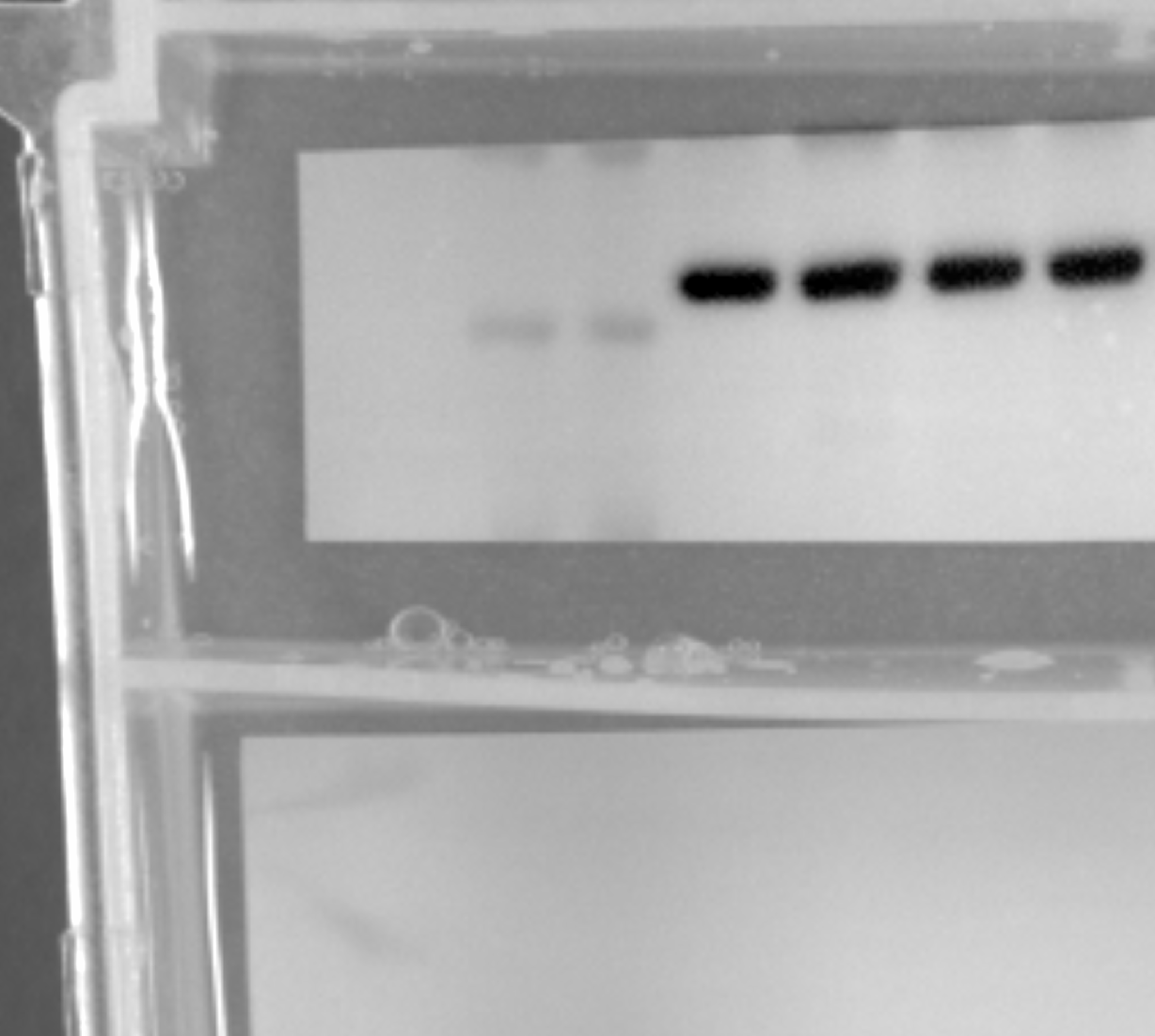

Supplement: Supplementary file 1 [file biomolecules-14-00591-s001.zip › WB_pictures_original/TSG101/actin-01-m+actin-01-used.tif]

## Slide 1
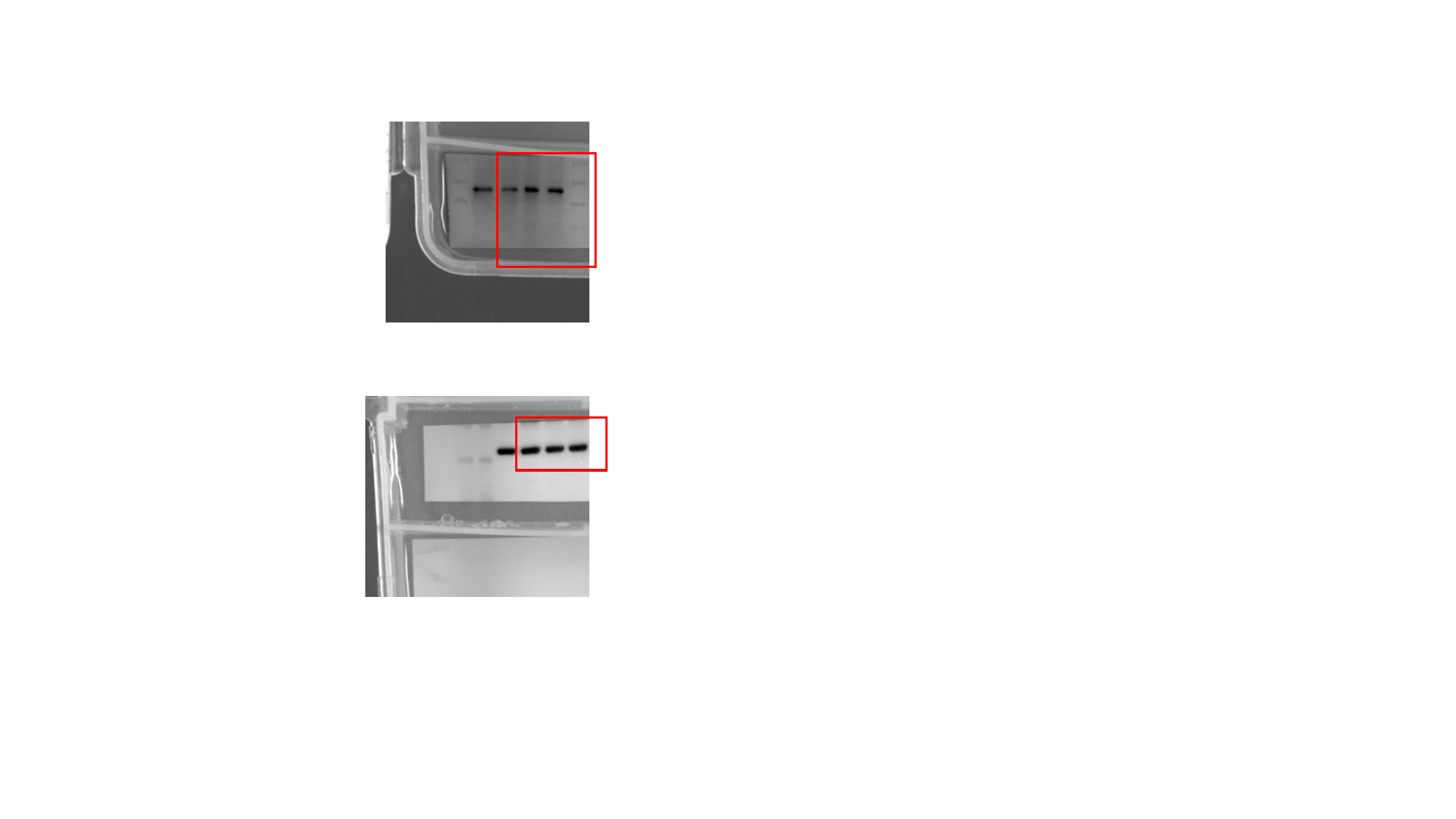

Supplement: Supplementary file 1 [file biomolecules-14-00591-s001.zip › WB_pictures_original/TSG101/Details_TSG101.pptx]

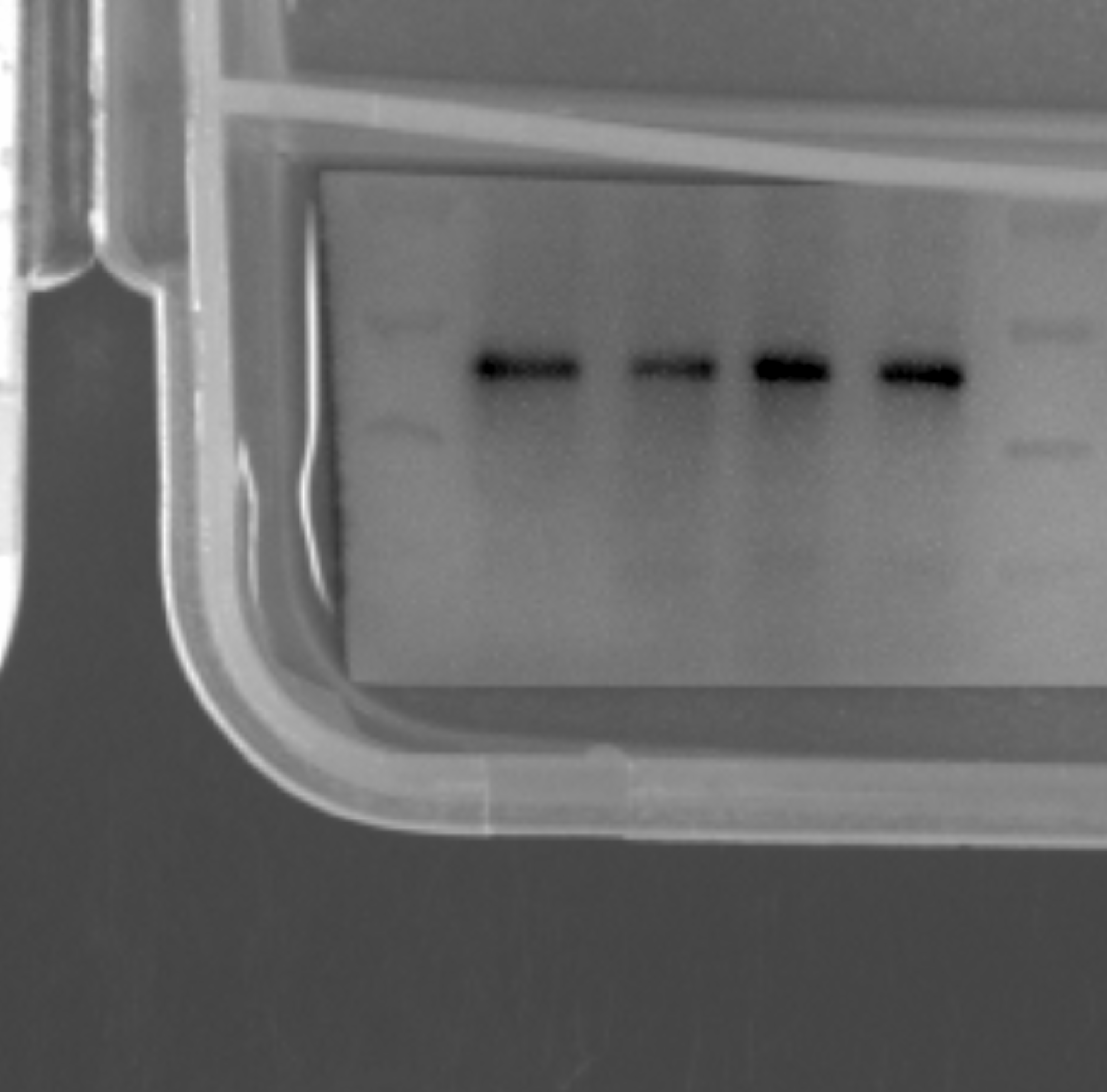

Supplement: Supplementary file 1 [file biomolecules-14-00591-s001.zip › WB_pictures_original/TSG101/tsg-02-01-m+tsg-02-01-used.tif]
